# Glucose-6-phosphate dehydrogenase regulates mitophagy by maintaining PINK1 stability

**DOI:** 10.1093/lifemeta/loae040

**Published:** 2024-12-13

**Authors:** Yik-Lam Cho, Hayden Weng Siong Tan, Jicheng Yang, Basil Zheng Mian Kuah, Nicole Si Ying Lim, Naiyang Fu, Boon-Huat Bay, Shuo-Chien Ling, Han-Ming Shen

**Affiliations:** Department of Physiology, Yong Loo Lin School of Medicine, National University of Singapore, Singapore 117593, Singapore; Department of Anatomy, Yong Loo Lin School of Medicine, National University of Singapore, Singapore 117594, Singapore; Department of Physiology, Yong Loo Lin School of Medicine, National University of Singapore, Singapore 117593, Singapore; Cancer and Stem Cell Biology Program, Duke-NUS Medical School, Singapore 169857, Singapore; Department of Physiology, Yong Loo Lin School of Medicine, National University of Singapore, Singapore 117593, Singapore; Department of Physiology, Yong Loo Lin School of Medicine, National University of Singapore, Singapore 117593, Singapore; Cancer and Stem Cell Biology Program, Duke-NUS Medical School, Singapore 169857, Singapore; Department of Anatomy, Yong Loo Lin School of Medicine, National University of Singapore, Singapore 117594, Singapore; NUS Centre for Cancer Research, Yong Loo Lin School of Medicine, National University of Singapore, Singapore 117599, Singapore; Department of Physiology, Yong Loo Lin School of Medicine, National University of Singapore, Singapore 117593, Singapore; Programs in Neuroscience and Behavioral Disorders, Duke-NUS Medical School, Singapore 169857, Singapore; Healthy Longevity Translational Research Programme, Yong Loo Lin School of Medicine, National University of Singapore, Singapore 117549, Singapore; Department of Physiology, Yong Loo Lin School of Medicine, National University of Singapore, Singapore 117593, Singapore; Faculty of Health Sciences, MOE Frontier Centre for Precision Oncology, University of Macau, Macao 999078, China

**Keywords:** G6PD, mitophagy, PINK1, ROS, NADPH, PPP

## Abstract

Glucose-6-phosphate dehydrogenase (G6PD) is the rate-limiting enzyme in the pentose phosphate pathway (PPP) in glycolysis. Glucose metabolism is closely implicated in the regulation of mitophagy, a selective form of autophagy for the degradation of damaged mitochondria. The PPP and its key enzymes such as G6PD possess important metabolic functions, including biosynthesis and maintenance of intracellular redox balance, while their implication in mitophagy is largely unknown. Here, via a whole-genome CRISPR-Cas9 screening, we identified that G6PD regulates PINK1 (phosphatase and tensin homolog [PTEN]-induced kinase 1)-Parkin-mediated mitophagy. The function of G6PD in mitophagy was verified via multiple approaches. G6PD deletion significantly inhibited mitophagy, which can be rescued by G6PD reconstitution. Intriguingly, while the catalytic activity of G6PD is required, the known PPP functions *per se* are not involved in mitophagy regulation. Importantly, we found a portion of G6PD localized at mitochondria where it interacts with PINK1. G6PD deletion resulted in an impairment in PINK1 stabilization and subsequent inhibition of ubiquitin phosphorylation, a key starting point of mitophagy. Finally, we found that G6PD deletion resulted in lower cell viability upon mitochondrial depolarization, indicating the physiological function of G6PD-mediated mitophagy in response to mitochondrial stress. In summary, our study reveals a novel role of G6PD as a key positive regulator in mitophagy, which bridges several important cellular processes, namely glucose metabolism, redox homeostasis, and mitochondrial quality control.

## Introduction

Macroautophagy (hereafter referred to as autophagy) is an evolutionarily conserved process in which cytosolic contents are sequestered into double-membrane vesicles known as autophagosomes and targeted for degradation by fusion with lysosomes [1, 2]. This process is vital for cellular function and survival, especially under nutrient stress [3–5]. Mitophagy is a form of selective autophagy involving the degradation of damaged mitochondria [6, 7]. In this process, damaged mitochondria are degraded via the autophagy-lysosome pathway to regulate mitochondrial turnover, which is critical for maintaining cellular homeostasis [8–10]. The molecular mechanisms of mitophagy have been extensively studied, and among them, the PINK1 (phosphatase and tensin homolog [PTEN]-induced kinase 1)-Parkin-dependent pathway is probably the most well-illustrated one. PINK1 is a protein kinase and Parkin is an E3 ubiquitin ligase, and their mutations have been linked to Parkinson’s disease (PD) [11–15]. Under basal or normal conditions, the newly synthesized full-length PINK1 is imported through the translocase of the outer membrane (TOM) complex located at the outer mitochondrial membrane (OMM) and the translocase of inner membrane (TIM) complex at the inner mitochondrial membrane (IMM), where it is cleaved by a mitochondrial processing peptidase (MPP), presenilins-associated rhomboid-like protein (PARL) [16]. The C-terminal of the cleaved PINK1 is then retro-translocated into the cytosol and degraded rapidly by the proteasome via the N-end rule pathway [17, 18]. As a result, PINK1 is maintained at a relatively low level in normal unstressed cells.

Upon mitochondrial damage with depolarization, mitochondrial import and cleavage of PINK1 is impaired, leading to its stabilization and activation at the OMM [16, 18–20]. Activated PINK1 then phosphorylates both ubiquitin (Ub) and Parkin at Serine-65 (S65), thus constituting a positive feedback loop in the PINK1-Parkin pathway [21–28]. Activated Parkin conjugates Ub chains on OMM proteins, leading to their subsequent proteasomal or autophagic degradation [29, 30]. However, despite the extensive studies done on this process, the upstream regulatory mechanisms of the PINK1-Parkin pathway in mitophagy remain to be elucidated.

Glucose is the most important energy source for cells. Upon uptake into the cell, glucose is processed in a series of biological reactions including glycolysis, tricarboxylic acid (TCA) cycle, and oxidative phosphorylation (OXPHOS), eventually resulting in the generation of energy in the form of ATP. In glycolysis, glucose is first converted to glucose-6-phosphate (G6P) by hexokinases. This step serves as the convergent point of other pathways using G6P, one of which is the pentose phosphate pathway (PPP) [31]. In the oxidative branch of the PPP, G6P is converted to ribulose-5-phosphate (R5P) in three irreversible reactions mediated by three consecutive enzymes, glucose-6-phosphate dehydrogenase (G6PD), 6-phosphogluconolactonase (PGLS or 6PGL), and phosphogluconate dehydrogenase (PGD or 6PGD) [31]. G6PD catalyzes the first rate-limiting step of the PPP, the conversion of G6P to 6-phosphogluconolactone, while also converting nicotinamide adenine dinucleotide phosphate (NADP^+^) to its reduced form, NADPH. In turn, NADPH is used as a reducing agent in the conversion of glutathione disulfide (GSSG) to glutathione (GSH). GSH serves as a key component of the cellular anti-oxidant system [32]. In addition to the production of NADPH as an important antioxidant, this pathway produces pentose phosphates for RNA and DNA synthesis [33]. In addition, deficiency or mutation in G6PD leads to G6PD deficiency (or favism), the most common human enzymopathy [34–36], partly due to certain mutations providing resistance against malaria [37–39]. However, the biological functions of G6PD in other cellular processes remain unclear.

In this study, via a whole-genome CRISPR-Cas9 screening, we first identified G6PD as one of the top hits as a positive regulator in PINK1-Parkin-mediated mitophagy. We then validated the role of G6PD in mitophagy and explored the molecular mechanism by which G6PD works via interacting with and stabilizing PINK1 upon mitochondrial depolarization. The results of this study thus reveal a novel function of G6PD in regulating mitophagy by targeting PINK1. Our study sheds new light on the intricate relationships between glucose metabolism, oxidative stress, and mitochondrial quality control, which are all closely implicated in human diseases such as neurodegenerative disorders.

## Results

### Generation of a HeLa cell line (HeLa triple-positive, HeLa 3+) with stable expression of mCherry-Parkin, mito-GFP, and miRFP-FLAG-Cas9

**Figure 1:**
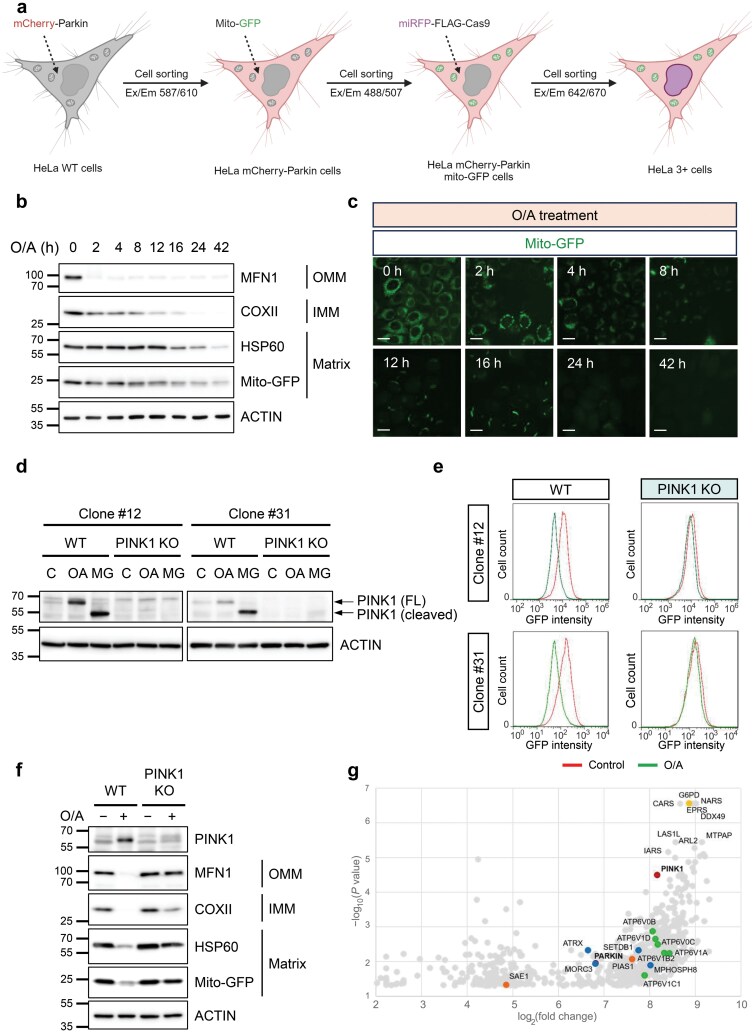
Generation of HeLa cell line expressing fluorescence reporter for mitochondrial content. (a) Schematic diagram of the screening process using HeLa 3+ cells. Briefly, the cell line was created by sequentially overexpressing mCherry-Parkin, mito-GFP, and miRFP-FLAG-Cas9 in that order using viral transfection methods. Cell sorting was done at each stage to isolate expressing cells. The eventual HeLa 3+ cell line was then subjected to lentiviral library transduction (~180,000 sgRNAs) and puromycin selection to isolate sgRNA-expressing cells. These cells were treated with O/A and subjected to FACS analysis using GFP as a readout for mitochondrial content. Indicated populations were collected and sent for sequencing to identify positive regulators of mitophagy. The process was repeated for clone #12 and clone #31 (*n* = 2). Created with BioRender.com. (b) Detection of mitochondrial protein levels. HeLa 3+ cells were treated with O/A (5 μmol/L and 1 μmol/L, respectively) for the indicated time points before analysis by SDS-PAGE and western blotting with the indicated antibodies. (c) Changes of mito-GFP intensity measured by live cell imaging. HeLa 3+ cells were treated with O/A (5 μmol/L and 1 μmol/L, respectively) for up to 42 h and images were taken at the indicated time points. Cells were imaged using a Leica fluorescence microscope. Scale bar: 20 μm. (d) PINK1 dynamics in HeLa 3+ cells. HeLa 3+ cells (clones #12 and #31) were treated with O/A (5 μmol/L and 1 μmol/L, respectively) or proteasome inhibitor MG132 (10 μmol/L) for 4 h. FL: full-length. (e) Reduction of mito-GFP measured by flow cytometry. Clone #12 and clone #31 of HeLa 3+ cells (WT and PINK1 KO) were treated with O/A (5 μmol/L and 1 μmol/L, respectively) for 42 h and subjected to flow cytometry analysis. *x*-axis, GFP intensity; *y*-axis, cell count. (f) Immunoblotting analysis of the clone #31 samples from (e). (g) Dot plot representing the hits falling within the analysis criteria. Red data points represent *PINK1* and *Parkin*. Blue data points represent genes involved in zinc finger gene regulation. Orange data points represent genes involved in SUMOylation. Green data points represent subunits of the vacuolar ATPase (V-ATPase). Yellow data point represents *G6PD*.

To facilitate the whole-genome CRISPR-Cas9 screening for mitophagy regulators, we first constructed a stable cell line in which HeLa cells were ectopically expressed with mCherry-Parkin, mito-GFP, and miRFP-FLAG-Cas9 (Fig. 1a). First, as HeLa cells do not contain endogenous Parkin, mCherry-Parkin was stably overexpressed. Second, Mito-GFP, which is composed of GFP tagged to mitochondrial cytochrome c oxidase subunit 8 (COX8) that is localized in the mitochondrial matrix [40], was used as a readout for the degradation of mitochondria, i.e. mitophagy. Finally, miRFP-FLAG-Cas9 was stably expressed to facilitate the usage of the CRISPR-Cas9 gene library. We thus named this HeLa cell line with stable expression of mito-GFP, mCherry-Parkin, and miRFP-FLAG-Cas9 as HeLa 3+ cells (Fig. 1a).

Next, we validated that HeLa 3+ cells were appropriate for mitophagy study, similar to the widely used HeLa YFP-Parkin cells. To do this, HeLa 3+ cells were treated with oligomycin A (electron transport chain complex V inhibitor) and antimycin A (complex Ⅲ inhibitor) (O/A) to induce mitochondrial depolarization to initiate mitophagy. As shown in Fig. 1b, O/A-treated HeLa 3+ cells followed a dynamic temporal pattern of mitochondrial protein degradation: first, the OMM protein mitofusin 1 (MFN1); then, the IMM protein COXII; and finally, the matrix protein HSP60. Notably, the degradation of mito-GFP follows the same pattern as HSP60 (Fig. 1b). To further confirm the data from immunoblotting analysis, we assessed mitochondrial clearance by live-cell imaging of mito-GFP and found a consistent temporal pattern of mito-GFP expression following O/A treatment (Fig. 1c). All these data indicate that the HeLa 3+ cells are suitable for studying mitophagy.

To ensure homogeneity in GFP expression, the parental HeLa 3+ population was subjected to single-cell sorting for the establishment of single-cell clones. Two clones, #12 and #31, were selected for further analysis. To further verify the usability of these two clones, we performed PINK1 knockout using CRISPR-Cas9. The deletion of PINK1 was confirmed by the absence of full-length PINK1 protein in cells treated with O/A, or the cleaved PINK1 in cells treated with MG132, a proteasome inhibitor to prevent the degradation of the cleaved PINK1 (Fig. 1d). Consistently, in both clones, treatment with O/A led to a significant reduction of GFP fluorescence intensity detected by flow cytometry (Fig. 1e), a feature to be used for the whole-genome screening described later. As expected, the deletion of PINK1 completely blocked O/A-induced mitophagy measured by the mito-GFP fluorescence intensity (Fig. 1e) and degradation of various mitochondrial proteins (Fig. 1f). With this validation, we moved on to use the HeLa 3+ cells for whole-genome CRISPR-Cas9 screening.

### Systematic identification of potential mitophagy regulators using whole-genome CRISPR-Cas9 screen

Two clones (#12 and #31) of the HeLa 3+ cells were independently transduced with a lentiviral library containing Human Two Plasmid Activity-Optimized CRISPR Knockout Library, which contains ~180,000 single-guide RNAs (sgRNAs) covering 18,000 genes in the human genome (~10 sgRNAs per gene) [41]. Details of the screening process are presented in the Materials and Methods section. Samples were then subjected to sgRNA enrichment analysis through next-generation sequencing (NGS) conducted by two vendors (Supplementary Fig. S1a and b). The two independent clones together with two rounds of sequencing produced four datasets, two from clone #12 (datasets #1 and #3) and two from clone #31 (datasets #2 and #4) (Supplementary Fig. S1b).

Analysis of the screening results was done using the datasets from clone #31, namely datasets #2 and #4. The overlap between these two datasets consisted of 263 overlapping genes (Supplementary Fig. S1c). Out of these genes, *PARK2* and *PINK1*, encoding Parkin and PINK1, respectively, were among the top hits, validating the effectiveness of this model to study PINK1-Parkin-mediated mitophagy. As expected, a majority of the top hits were largely related to housekeeping processes required for basic cell survival and homeostasis, such as transcription and translation factors. In addition, pathway analysis was conducted using Reactome (Supplementary Fig. S1d) [42]. The appearance of PINK1-Parkin-mediated mitophagy validated once again the effectiveness of using this model to study this pathway of mitophagy. In addition, several other gene groups were revealed, involving the cell cycle, ribosomal regulation, and transcription and translation. It is worthy to note that besides these housekeeping processes, only two other pathways were identified in the analysis, namely TP53 regulation and glucose metabolism. A dot plot of the genes meeting these criteria is shown in Fig. 1g, with significant genes and gene groups highlighted.

### Identification of G6PD as a positive regulator of mitophagy

**Figure 2:**
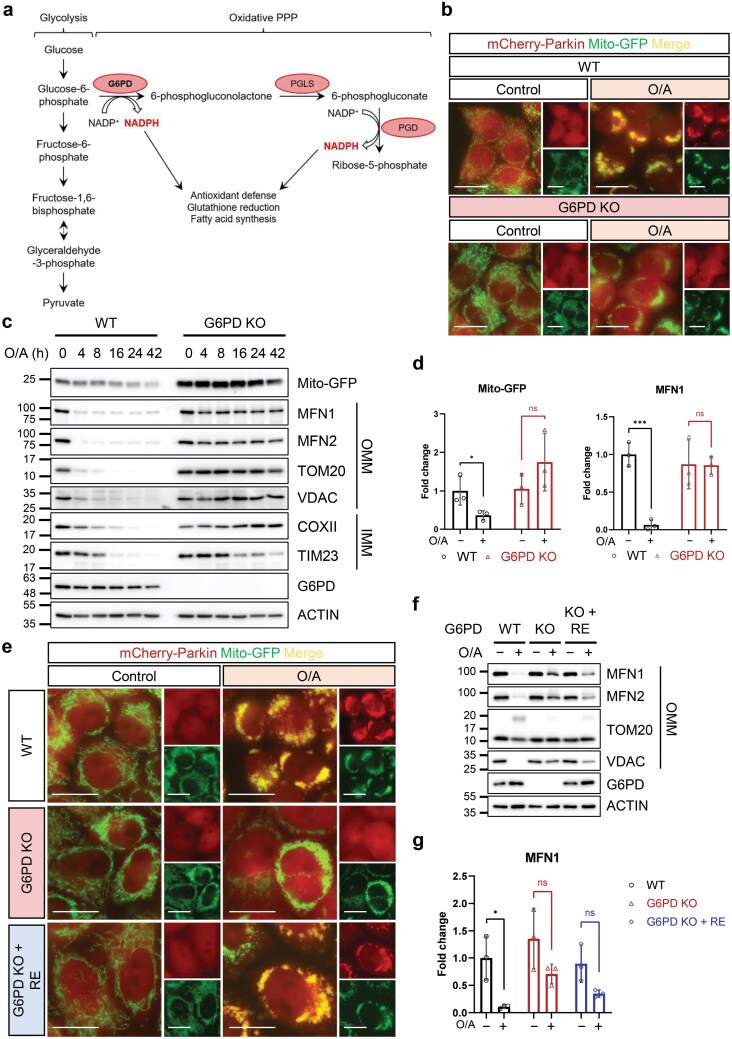
Identification of G6PD as a positive regulator of mitophagy. (a) The PPP. G6PD is the rate-limiting enzyme converting G6P to 6-phosphogluconolactone. (b) Changes in mCherry-Parkin translocation measured by live cell imaging. WT and G6PD KO HeLa 3+ cells were treated with O/A (5 μmol/L and 1 μmol/L, respectively) for 4 h. Live images were taken with a Leica fluorescence microscope. Scale bar: 20 μm. (c) Detection of mitochondrial protein levels by immunoblotting analysis. WT and G6PD KO HeLa 3+ cells were treated with O/A (5 μmol/L and 1 μmol/L, respectively) for indicated time points and subjected to immunoblotting analysis. (d) Quantification of mito-GFP and MFN1 levels in lysates after 42-h O/A treatment as seen in (c). Fold change was calculated using wild-type, untreated cells as the baseline. (e) Changes in mCherry-Parkin translocation measured by live cell imaging. G6PD KO HeLa 3+ cells were transfected with WT G6PD for 24 h. Cells were treated with O/A (5 μmol/L and 1 μmol/L, respectively) for 4 h. Images were taken with a Leica fluorescence microscope. Scale bar: 20 μm. (f) Detection of mitochondrial protein levels. G6PD KO HeLa 3+ cells were transfected with WT G6PD for 24 h. Cells were then treated with O/A (5 μmol/L and 1 μmol/L, respectively) for 4 h and subjected to immunoblotting analysis. RE: reconstituted. (g) Quantification of MFN1 levels in G6PD KO and reconstituted cell lysates as seen in (f). Fold change was calculated using wild-type, untreated cells as the baseline. RE: reconstituted. Data in (d) and (g) are presented as mean ± SD of three independent experiments. ns: not significant; ^*^P < 0.05; ^***^P < 0.001.

G6PD is known as one of the essential enzymes in the PPP [31] (Fig. 2a). Interestingly, among the top gene hits in datasets #2 and #4, *G6PD* was identified with high confidence (Fig. 1g; Supplementary Fig. S1e). This finding, together with many other genes related to glucose metabolism identified in the Reactome analysis (Supplementary Fig. S1d), led us to further investigate this gene in the regulation of mitophagy.

To validate the results from this CRISPR-Cas9 screen, we first established a stable cell line with deletion of G6PD in HeLa 3+ cells. Two independent sgRNAs were utilized, and the cells created with sgRNA #1 were used for further study, given the similar degree of G6PD KO between the two KO cell lines (Supplementary Fig. S2a). Indeed, Parkin translocation to the mitochondria was inhibited in G6PD KO HeLa 3+ cells compared to wild-type cells treated with O/A for 4 h (Fig. 2b; Supplementary Fig. S2b). Correspondingly, there is an inhibition of mitochondrial protein degradation in cells treated with O/A for up to 42 h (Fig. 2c and d), indicating not just a delay in mitophagy induction, but an inhibition of mitophagy altogether. This phenotype is similarly seen in G6PD KO cells created using sgRNA #2 (Supplementary Fig. S2c and d), as well as in clone #12 (Supplementary Fig. S2e), indicating that these observations are not specific to the sgRNA or clone used. Furthermore, an inhibition of mitochondrial protein degradation was also observed in carbonyl cyanide m-chlorophenyl hydrazone (CCCP)-treated G6PD KO cells, suggesting that G6PD may be a general regulator of mitophagy (Supplementary Fig. S2f). Importantly, reconstitution of the wild-type (WT) G6PD into G6PD KO cells to an activity level comparable to the endogenous level markedly restored Parkin mitochondrial translocation upon O/A treatment (Fig. 2e; Supplementary Fig. S2g and h) and resulted in a recovery in mitochondrial protein degradation in comparison to the WT cells (Fig. 2f and g). Taken together, the data demonstrate that G6PD is a positive regulator of mitophagy.

### The involvement of G6PD in mitophagy is independent of its known role in the PPP

G6PD is the first enzyme in the PPP that mediates the conversion of G6P to 6-phosphogluconolactone, in which NADPH is produced [43] (Fig. 2a). NADPH acts as an antioxidant, which is particularly important to mitochondrial function based on the fact that mitochondria are the most important source of ROS production [44, 45].

**Figure 3:**
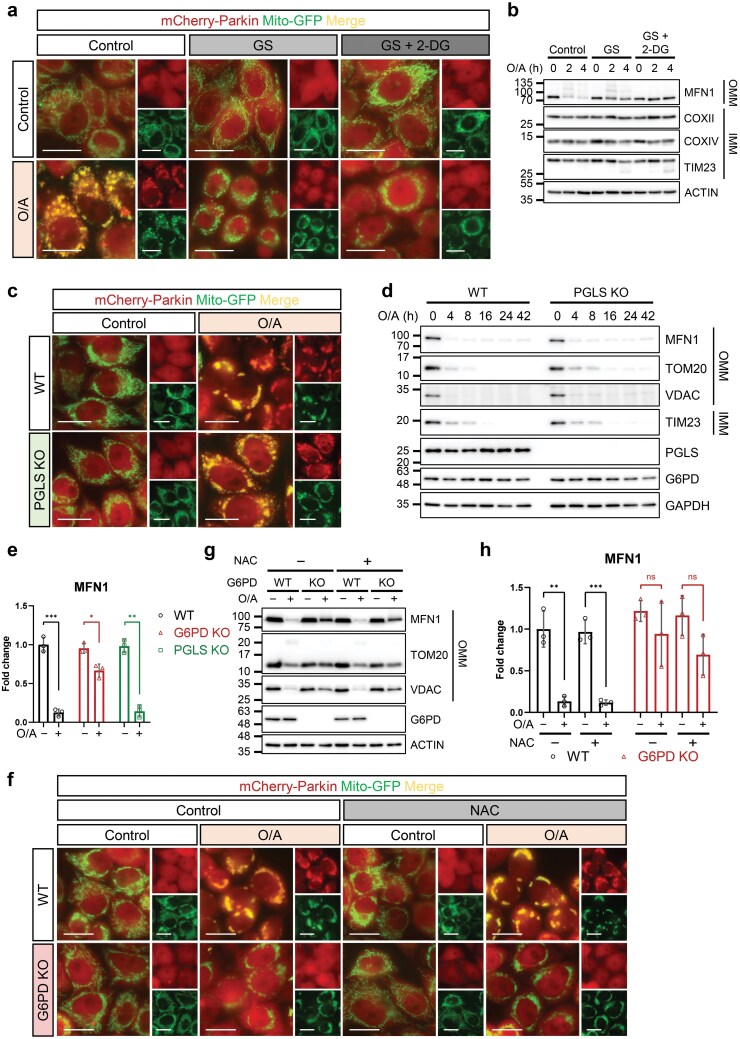
The involvement of G6PD in mitophagy is independent of its known role in the PPP. (a) Changes in mCherry-Parkin translocation measured by live cell imaging. HeLa 3+ cells were treated with glucose starvation media (GS) or glucose starvation media supplemented with 5 mmol/L 2-DG (GS + 2-DG) for 2 h. Cells were then treated with O/A (5 μmol/L and 1 μmol/L, respectively) for 4 h, and live images were taken with a Leica fluorescence microscope. Scale bar: 20 μm. (b) SDS-PAGE and western blotting analysis of cells treated as in (a) and collected at indicated time points. (c) Changes in mCherry-Parkin translocation measured by live cell imaging. PGLS KO HeLa 3+ cells were created using the CRISPR-Cas9 system. Cells were treated with O/A (5 μmol/L and 1 μmol/L, respectively) for 4 h and live cell fluorescence imaging was conducted. Scale bar: 20 μm. (d) Detection of mitochondrial protein levels. PGLS KO HeLa 3+ cells were treated with O/A (5 μmol/L and 1 μmol/L, respectively) for the indicated time points. Lysates were subjected to SDS-PAGE and western blotting analysis. (e) Quantification of MFN1 levels in cells treated with O/A (5 μmol/L and 1 μmol/L, respectively) for 4 h. Fold change was calculated using wild-type, untreated cells as the baseline. (f) Changes in mCherry-Parkin translocation measured by live cell imaging. HeLa 3+ cells (WT and G6PD KO) were treated with NAC (10 mmol/L) and O/A (5 μmol/L and 1 μmol/L, respectively) for 4 h. Cells were subjected to live cell imaging. Scale bar: 20 μm. (g) Cells were treated as in (f) and subjected to SDS-PAGE and western blotting analysis. (h) Quantification of MFN1 levels in O/A-treated cells as seen in (g). Fold change was calculated using wild-type, untreated cells as the baseline. Data in (e) and (h) are presented as mean ± SD of three independent experiments. ns: not significant; ^*^P < 0.05; ^**^P < 0.01; ^***^P < 0.001.

We first investigated whether the regulatory role of G6PD in mitophagy is dependent on the PPP. To this end, we manipulated the PPP by using glucose starvation and 2-deoxyglucose (2-DG). Glucose starvation will abolish both glycolysis and PPP, while 2-DG, a glucose analog, is unable to be processed through glycolysis but can be metabolized through the PPP, thus acting as a mimetic of glucose starvation while maintaining the PPP [46, 47]. Consistent with an earlier report [48], glucose starvation inhibited mitophagy, as evidenced by the blockage of Parkin translocation (Fig. 3a; Supplementary Fig. S3a) and mitochondrial protein degradation (Fig. 3b). Interestingly, addition of 2-DG into the glucose-free medium failed to affect Parkin translocation and mitochondrial protein degradation in WT HeLa 3+ cells treated with O/A (Fig. 3a and b; Supplementary Fig. S3a), suggesting that the PPP activation in cells supplemented with 2-DG is unable to rescue mitophagy in cells under glucose starvation. The effects of glucose starvation were verified by the levels of phosphorylated AMP-activated protein kinase (p-AMPK) and phosphorylated acetyl-CoA carboxylase (p-ACC) (Supplementary Fig. S3b). To further investigate the role of the PPP in mitophagy, we deleted the second enzyme of the PPP, PGLS (or 6PGL) using the CRISPR-Cas9 system in HeLa 3+ cells. In PGLS KO cells, Parkin translocation and mitochondrial protein degradation were comparable to WT cells (Fig. 3c−e; Supplementary Fig. S3c). Therefore, the impairment of mitophagy in G6PD-deficient cells is unlikely to be caused by a dysfunctional PPP, and G6PD likely exerts a regulatory effect on mitophagy independent of its role in the PPP. Nevertheless, since we did not measure any metabolic intermediates of the PPP, these results cannot exclude the possibility that some metabolic intermediates may be involved in mitophagy regulation.

A vital role of G6PD in the PPP is to produce NADPH, which in turn is involved in the conversion of GSSG to GSH, an important process in antioxidant defense [49]. Thus, the lack of G6PD, along with the damage to the electron transport chain (ETC) caused by O/A treatment, may cause an overwhelming level of ROS in the cell, resulting in perturbations in mitophagy activation. Interestingly, treatment with N-acetylcysteine (NAC), an antioxidant, was unable to recover Parkin translocation (Fig. 3f; Supplementary Fig. S3d) or mitochondrial protein degradation (Fig. 3g and h), even though this treatment reduced oxidative stress as evidenced by reduced phosphorylated c-Jun amino (N)-terminal kinase (p-JNK) (Supplementary Fig. S3e). These data further suggest that the defective mitophagy in G6PD KO cells is independent of the PPP and associated oxidative stress.

### G6PD catalytic activity is required for functional mitophagy

**Figure 4:**
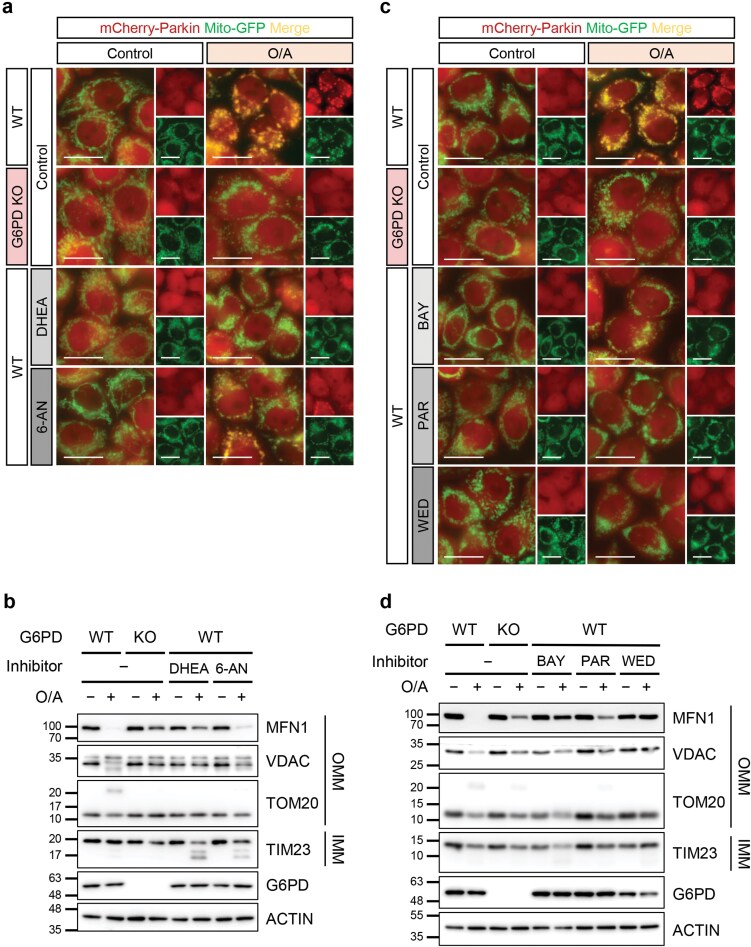
G6PD catalytic activity is required for functional mitophagy. (a) mCherry-Parkin translocation visualized by live cell imaging. HeLa 3+ cells were pretreated with G6PD inhibitors DHEA (500 μmol/L) or 6-AN (1 mmol/L) for 1 h before O/A (5 μmol/L and 1 μmol/L, respectively) treatment for 2 h. Cells were subjected to live cell imaging. G6PD KO cells were used as a negative control. Scale bar: 20 μm. (b) Detection of mitochondrial protein levels. HeLa 3+ cells were treated as in (a). Lysates were subjected to SDS-PAGE and immunoblotting analysis. G6PD KO cells were used as a negative control. (c) Detection of Parkin translocation with live cell imaging. HeLa 3+ cells were pretreated with Bay-11-7082 (BAY, 10 μmol/L), parthenolide (PAR, 25 μmol/L), or wedelolactone (WED, 50 μmol/L) for 1 h, followed by O/A (5 μmol/L and 1 μmol/L, respectively) treatment for 2 h. G6PD KO cells were used as a negative control. Scale bar: 20 μm. (d) Detection of mitochondrial protein levels. Cells were treated as in (c) and lysates were immunoblotted with indicated antibodies. G6PD KO cells were used as a negative control.

While the canonical G6PD function in PPP does not affect mitophagy regulation, we next examined whether the catalytic ability of G6PD itself is important in its regulatory role in mitophagy. To investigate this possibility, we utilized several G6PD chemical inhibitors, including a steroidal noncompetitive inhibitor Dehydroepiandrosterone (DHEA) [50] and a small-molecule competitive inhibitor 6-aminonicotinamide (6-AN) [51]. Treatment of HeLa 3+ cells with these inhibitors resulted in an inhibition of Parkin translocation (Fig. 4a; Supplementary Fig. S4a) and mitochondrial protein degradation induced by O/A treatment (Fig. 4b). Similarly, the treatments of HeLa 3+ cells with another three known G6PD inhibitors, Bay-11-7082, parthenolide, and wedelolactone [52, 53], produced similar results (Fig. 4c and d; Supplementary Fig. S4b). The absence of TIM23 degradation observed in Fig. 4b and d is due to the short time of O/A treatment.

To further investigate the involvement of the catalytic activity of G6PD, we examined the effects of a clinical mutant of G6PD on mitophagy, namely the Bangkok noi (BN) variant of G6PD. The BN mutant was shown in a previous study to have a lower substrate binding affinity for both G6P and NADP^+^ due to a mutation in amino acid residue 502 affecting the NADP^+^ binding pocket in the secondary structure [54]. Reconstitution of the BN mutant in G6PD KO cells did not recover Parkin translocation to the mitochondria to the same extent as the reconstitution of WT G6PD (Supplementary Fig. S4c). Taken together, although the PPP is not directly involved in the regulation of mitophagy by G6PD, the catalytic ability of this enzyme appears to be important in this process.

### G6PD is involved in PINK1 stabilization and Ub phosphorylation

**Figure 5:**
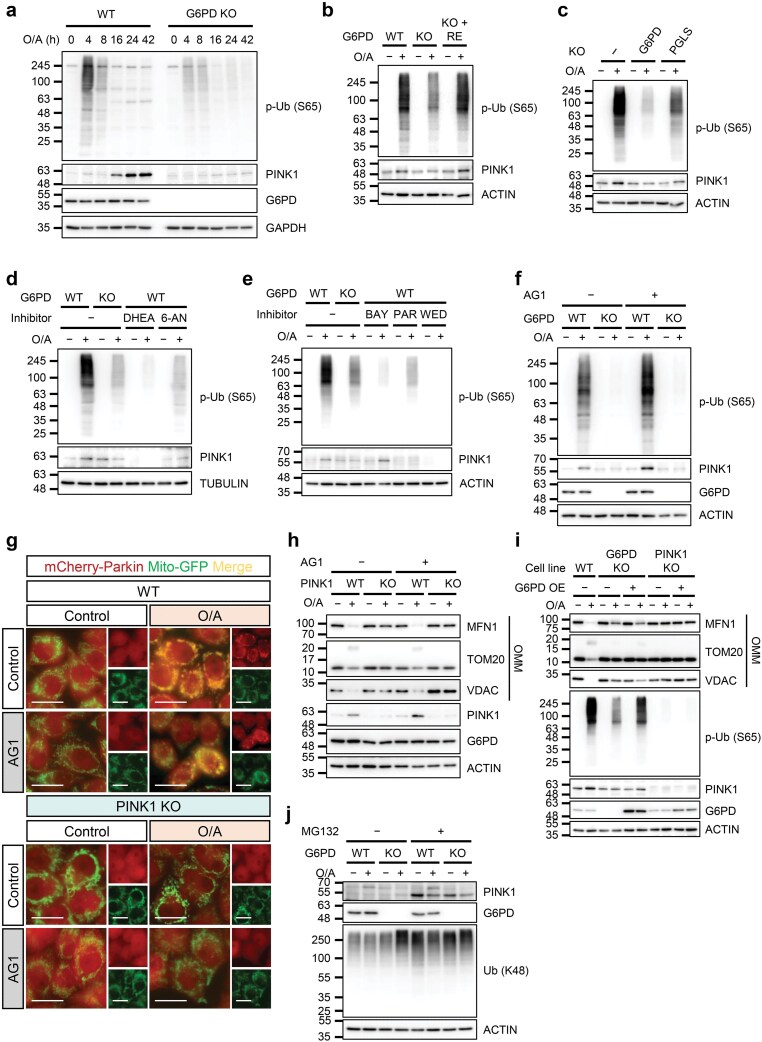
G6PD is involved in PINK1 stabilization and ubiquitin phosphorylation. (a−f) Detection of p-Ub and PINK1 protein levels by immunoblotting analysis. (a) WT and G6PD KO HeLa 3+ cells were treated with O/A (5 μmol/L and 1 μmol/L, respectively) for the indicated time points and subjected to immunoblotting analysis. (b) G6PD KO HeLa 3+ cells were transfected with WT G6PD for 24 h. Cells were then treated with O/A (5 μmol/L and 1 μmol/L, respectively) for 4 h and subjected to immunoblotting analysis. RE: reconstituted. (c) WT, G6PD KO, and PGLS KO HeLa 3+ cells were treated with O/A (5 μmol/L and 1 μmol/L, respectively) for 4 h. Lysates were subjected to immunoblot analysis. (d) HeLa 3+ cells were pretreated with G6PD inhibitors DHEA (500 μmol/L) or 6-AN (1 mmol/L) for 1 h before O/A (5 μmol/L and 1 μmol/L, respectively) treatment for 2 h. G6PD KO cells were used as a negative control. (e) HeLa 3+ cells were pretreated with BAY (10 μmol/L), PAR (25 μmol/L), or WED (50 μmol/L) for 1 h followed by O/A (5 μmol/L and 1 μmol/L, respectively) treatment for 2 h. G6PD KO cells were used as a negative control. Loading control is shared with Fig. 4d. (f) WT and G6PD KO HeLa 3+ cells were pretreated with AG1 (20 μmol/L) for 1 h before O/A (5 μmol/L and 1 μmol/L, respectively) treatment for 4 h. (g) Visualization of mCherry-Parkin translocation by live cell imaging. HeLa 3+ (WT and PINK1 KO) cells were pretreated with AG1 (20 μmol/L) for 1 h before O/A (5 μmol/L and 1 μmol/L, respectively) treatment for 2 h. Images were taken using a Leica fluorescence microscope. Scale bar: 20 μm. (h) Detection of mitochondrial protein levels by immunoblotting. HeLa 3+ (WT and PINK1 KO) cells were pretreated with AG1 (20 μmol/L) for 1 h before O/A (5 μmol/L and 1 μmol/L, respectively) treatment for 4 h. Lysates were subjected to immunoblotting analysis with the indicated antibodies. (i) Mitochondrial protein levels in G6PD-overexpressing cells. G6PD KO and PINK1 KO HeLa 3+ cells were transfected with WT G6PD for 24 h. Cells were treated with O/A (5 μmol/L and 1 μmol/L, respectively) for 4 h before lysis and immunoblotting analysis. (j) Cleaved PINK1 levels observed by immunoblotting analysis. HeLa 3+ (WT and G6PD KO) cells were pretreated with MG132 (10 μmol/L) for 1 h before being treated with O/A (5 μmol/L and 1 μmol/L, respectively) for 4 h. Lysates were immunoblotted with the indicated antibodies.

Since the PPP does not appear to play a role in G6PD-mediated mitophagy but the enzymatic ability of G6PD is involved, we hypothesized that G6PD may play a novel role in mediating mitophagy directly. Indeed, full-length PINK1 was not stabilized in G6PD KO cells under O/A treatment (Fig. 5a; Supplementary Fig. S5a) or CCCP treatment (Supplementary Fig. S5b). Correspondingly, there was no phospho-Ub (p-Ub) detected in these cells (Fig. 5a; Supplementary Fig. S5a and b). Again, these phenotypes were sustained over up to 42 h, indicating that mitophagy is not just delayed, but persistently inhibited. Of note, in WT cells, p-Ub levels increased at 4 h and began to decrease over longer time points, while PINK1 stabilization is sustained up to 42 h. This could be explained by the dephosphorylation of p-Ub by PTEN-long (PTEN-L) [55] or the degradation of p-Ub along with the mitochondria during mitophagy. Importantly, p-Ub levels and PINK1 stabilization were recovered in G6PD KO cells reconstituted with WT G6PD (Fig. 5b). In contrast, supplementation of 2-DG did not recover PINK1 and p-Ub levels (Supplementary Fig. S3b), and knockout of PGLS did not inhibit PINK1 stabilization or ubiquitin phosphorylation (Fig. 5c; Supplementary Fig. S5c), consistent with the notion that the effect of G6PD on PINK1 is independent of the PPP.

To further examine the relationship between G6PD and PINK1, we tested the changes of PINK1 in cells treated with G6PD inhibitors (Fig. 5d and e) and the catalytically inactive BN mutant of G6PD (Supplementary Fig. S5d). Under these conditions, PINK1 failed to accumulate and Ub phosphorylation was impaired. This suggests that the catalytic ability of G6PD contributes to PINK1 stabilization and kinase activity, working upstream of PINK1. To further establish this connection, a G6PD activator AG1 was used. Treatment with AG1 enhanced PINK1 stabilization and Ub phosphorylation after O/A treatment in WT cells, but not in G6PD KO cells (Fig. 5f; Supplementary Fig. S5e). Furthermore, AG1 did not recover Parkin translocation (Fig. 5g; Supplementary Fig. S5f) or mitochondrial protein degradation (Fig. 5h) in O/A-treated PINK1 KO cells, indicating that the regulatory effect of G6PD on mitophagy is through PINK1. Consistently, overexpression of WT G6PD in PINK1 KO cells did not recover mitochondrial protein degradation or Ub phosphorylation (Fig. 5i), further indicating that G6PD works upstream of PINK1 in the regulation of mitophagy.

It is well-established that in unstressed cells, cleaved PINK1 is quickly degraded by the proteasome via the N-end rule [18]. Thus, we also tested the effect of MG132, a proteasome inhibitor, and found that in G6PD KO cells, there was no accumulation of full-length PINK1 under O/A treatment even though there was still PINK1 production as observed by the presence of cleaved PINK1 (Fig. 5j; Supplementary Fig. S5g). This suggests that G6PD facilitates the stabilization of full-length PINK1 during mitophagy at the protein level as opposed to at a transcriptional level. Taken together, these results indicate that G6PD operates upstream of PINK1 in mitophagy and acts to facilitate the stabilization and activation of the full-length PINK1 in a way that is independent of the PPP but dependent on its catalytic ability.

### G6PD interacts with the mitophagy machinery

After uncovering the effect of G6PD on full-length PINK1, we next investigated whether there was an interaction between G6PD and PINK1. G6PD is known to be a cytosolic protein, although studies have revealed that G6PD also localizes to ribosomes and peroxisomes [56], as well as to the mitochondria [57, 58]. It has also been shown that mitochondrial G6PD is important for maintaining mitochondrial NADPH pools to provide antioxidant power in high glucose conditions [59].

**Figure 6:**
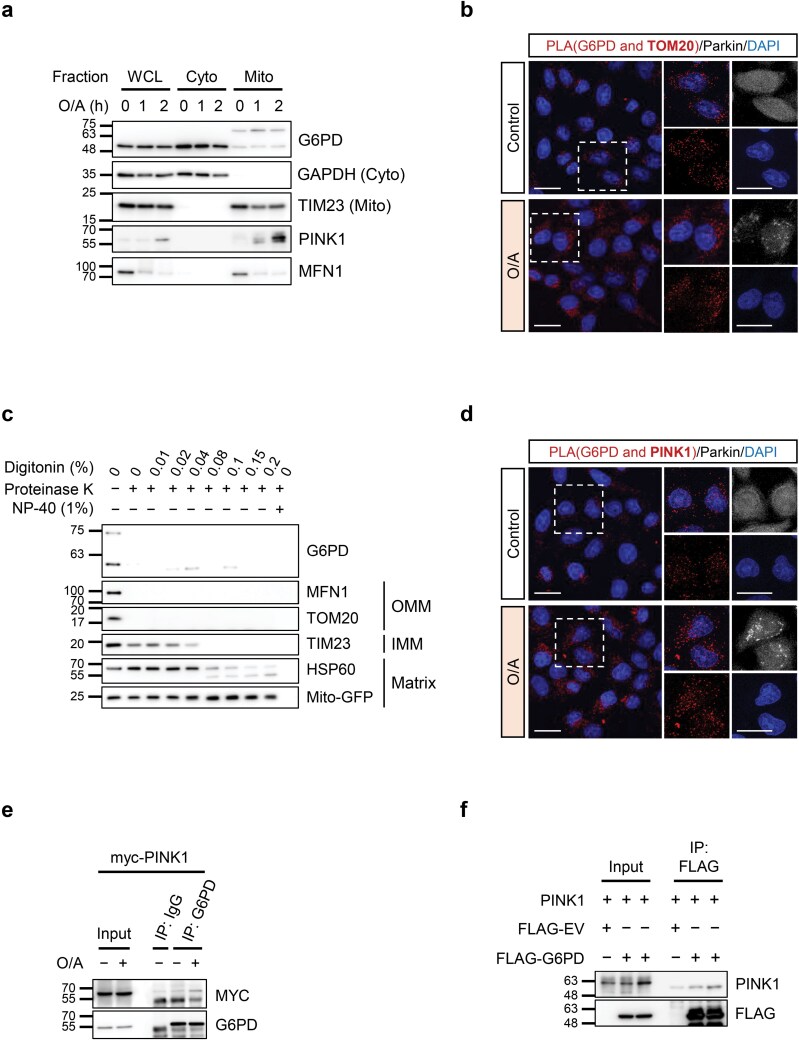
G6PD interacts with the mitophagy machinery. (a) Mitochondrial G6PD levels detected by immunoblotting analysis. HeLa 3+ cells were treated with O/A (5 μmol/L and 1 μmol/L, respectively) for 1 h or 2 h. Cells were subjected to fractionation by sucrose gradient centrifugation and immunoblotted with the indicated antibodies. Sample loading was standardized to the whole cell lysate. GAPDH, cytosolic marker; TIM23, mitochondrial marker. WCL, whole cell lysate; Cyto, cytosol; Mito, mitochondria. (b) PLA performed on YFP-Parkin HeLa cells. Cells were treated with O/A (5 μmol/L and 1 μmol/L, respectively) for 90 min. Assay was performed using G6PD and TOM20 primary antibodies. Red, PLA signal; gray (pseudocoloured), YFP-Parkin; blue, DAPI. Scale bar: 20 μm. (c) Visualization of G6PD mitochondrial localization by proteinase K protection assay. HeLa 3+ cells were treated with O/A (5 μmol/L and 1 μmol/L, respectively) for 90 min and subjected to fractionation by sucrose gradient centrifugation. The mitochondrial fraction was divided and treated with the indicated concentrations of digitonin and proteinase K. Samples were then subjected to immunoblotting analysis. (d) PLA performed on YFP-Parkin HeLa cells. Cells were treated with O/A (5 μmol/L and 1 μmol/L, respectively) for 90 min. Assay was performed using G6PD and PINK1 primary antibodies. Red, PLA signal; gray (pseudocoloured), YFP-Parkin; blue, DAPI. Scale bar: 20 μm. (e) Immunoprecipitation of overexpressed myc-PINK1 with endogenous G6PD. HeLa 3+ cells were transfected with myc-PINK1 for 24 h. Cells were treated with O/A (5 μmol/L and 1 μmol/L, respectively) for 90 min and lysed in IP lysis buffer. Lysates were subjected to IP with agarose-conjugated G6PD antibody and blotted with the indicated antibodies. (f) *In vitro* pull-down assay between G6PD and PINK1. HeLa mCherry-Parkin cells were transfected with FLAG-tagged G6PD. FLAG-G6PD protein was isolated using FLAG M2 beads and incubated with recombinant human PINK1 protein. Samples were subjected to SDS-PAGE and western blotting analysis to visualize pull-down of PINK1.

To address whether G6PD is localized to the mitochondria to regulate PINK1-Parkin-mediated mitophagy in our model, we conducted a cellular fractionation assay to isolate mitochondria and found a substantial amount of G6PD in the mitochondrial fraction under both basal and O/A-treated conditions (Fig. 6a). In addition, it was observed that there was a modified, higher molecular weight form of G6PD that was found only in the mitochondrial fraction (Fig. 6a). This modified form of G6PD is unlikely to be a dimer (~110 kDa). Instead, it could be a post-translationally modified G6PD, likely a phosphorylated or ubiquitinated form of G6PD, suggesting that post-translational modification of G6PD may be required for mitophagy regulation.

G6PD mitochondrial localization was further verified *in situ* by proximity ligation assay (PLA), the result showing that G6PD is in the proximity of OMM protein TOM20 (Fig. 6b). In addition, to investigate the intra-mitochondrial localization of G6PD, the mitochondrial fraction of HeLa 3+ cells was subjected to a proteinase K protection assay. As expected, both the regular and high molecular weight G6PD were found in the control fraction without digitonin or proteinase K treatment. However, G6PD was degraded by proteinase K alone without membrane disruption by digitonin, indicating that G6PD localizes to the OMM similarly to TOM20 and MFN1 (Fig. 6c).

Given the observed localization of G6PD at the mitochondria, there was a possibility that G6PD may interact with the PINK1-Parkin machinery during mitophagy. Indeed, G6PD showed positive signals with PINK1 using PLA (Fig. 6d). In addition, overexpressed myc-PINK1 co-immunoprecipitated with endogenous G6PD (Fig. 6e). Further examination of this interaction was done by purifying FLAG-G6PD from mCherry-Parkin-HeLa cells followed by incubation with recombinant human PINK1 protein. In this case, G6PD showed an interaction with recombinant PINK1 protein (Fig. 6f). Altogether, data from this part of our study suggest that G6PD regulates PINK1 protein stability and kinase activity via an association with PINK1 at the OMM.

To determine which domain of G6PD interacts with PINK1, we created three different myc-tagged truncation mutants of G6PD, each lacking one of the known domains of G6PD (Supplementary Fig. S6a), and overexpressed them in HeLa YFP-Parkin cells. The interaction between each of these mutants and PINK1 was analyzed via PLA (Supplementary Fig. S6b). G6PD lacking its dimerization domain (myc-G6PD-ΔD) or NADP^+^-binding domain (myc-G6PD-ΔN) retained binding ability to PINK1 as evidenced by the positive PLA signals with PINK1 (Supplementary Fig. S6b). On the other hand, G6PD lacking the C-terminal domain (myc-G6PD-ΔC) did not exhibit any binding to PINK1 (Supplementary Fig. S6b), suggesting that the C-terminal region of G6PD is sufficient and necessary for interaction with PINK1.

As expected, reconstitution of myc-G6PD-ΔC in G6PD KO cells was not able to recover mitophagy phenotypes (Supplementary Fig. S6c), indicating that the interaction between G6PD and PINK1 is essential for mitophagy. Interestingly, although both myc-G6PD-ΔD and myc-G6PD-ΔN were able to interact with PINK1, only reconstitution of myc-G6PD-ΔN in G6PD KO cells was able to recover mitophagy (Supplementary Fig. S6c), suggesting that although the C-terminal region is required for interaction with PINK1, the dimerization domain within it specifically, or dimer formation of G6PD, is important for the regulation of PINK1 and hence activation of mitophagy.

### G6PD-mediated mitophagy is required to maintain cell viability during mitochondrial stress

**Figure 7:**
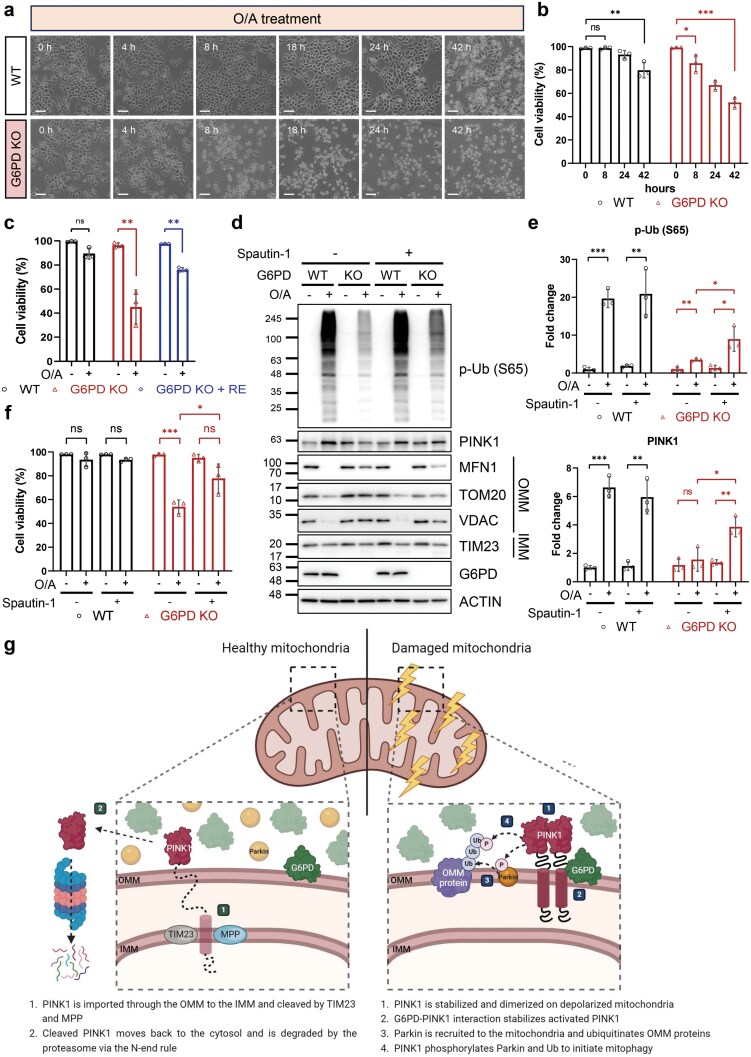
G6PD-mediated mitophagy is required to maintain cell viability during mitochondrial stress. (a) Detection of cell viability using bright field imaging. WT and G6PD KO HeLa 3+ cells were treated with O/A (5 μmol/L and 1 μmol/L, respectively) for indicated time points and subjected to bright field imaging. Scale bar: 200 μm. (b) Quantification of the data from (a). At least 500 cells per sample, for three independent samples, were counted by trypan blue exclusion. Only data from 0, 8, 24, and 42 h time points are presented. (c) Cell viability of G6PD KO HeLa 3+ cells reconstituted with WT G6PD. G6PD KO HeLa 3+ cells were transfected with WT G6PD for 24 h. Cells were then treated with O/A (5 μmol/L and 1 μmol/L, respectively) for 24 h. At least 500 cells per sample, for three independent samples, were counted by trypan blue exclusion. RE: reconstituted. (d) Protein levels observed by immunoblotting analysis. HeLa 3+ (WT and G6PD KO) cells were pretreated with Spautin-1 (10 μmol/L) for 1 h before being treated with O/A (5 μmol/L and 1 μmol/L, respectively) for 4 h. Lysates were subjected to SDS-PAGE and western blotting analysis with the indicated antibodies. (e) Quantification of p-Ub (top) and PINK1 (bottom) levels as seen in (d). Fold change was calculated using wild-type, untreated cells as the baseline. (f) Cell viability of G6PD KO cells treated with Spautin-1. HeLa 3+ (WT and G6PD KO) cells were pretreated with Spautin-1 (10 μmol/L) for 1 h before being treated with O/A (5 μmol/L and 1 μmol/L, respectively) for 16 h. At least 500 cells per sample, for three independent samples, were counted by trypan blue exclusion. The data in (b), (c), (e), and (f) are presented as mean ± SD of three independent experiments. ns: not significant. ^*^P < 0.05; ^**^P < 0.005; ^***^P < 0.001. (g) Proposed mechanism of regulation of mitophagy by G6PD. In healthy mitochondria, PINK1 is cleaved by TIM23 and MPP at the IMM and degraded by the proteasome. In damaged mitochondria, i.e. under O/A treatment, PINK1 is stabilized on the OMM and phosphorylates Parkin and Ub. G6PD interacts with this mitochondrial PINK1 to facilitate its stabilization and activity. Created with BioRender.com.

G6PD plays an important role in the cellular response to oxidative stress by providing reducing power in the form of NADPH [60]. NADPH not only acts to maintain redox homeostasis but also functions as a co-factor for many antioxidant enzymes [44]. O/A treatment, on the other hand, contributes to oxidative stress due to the inhibition of the ETC and OXPHOS. Therefore, the absence of G6PD on the basis of O/A treatment may cause more severe oxidative stress due to detrimental ROS accumulation. Indeed, treatment of G6PD KO cells with O/A caused evident cell death as early as 8 h, compared to WT cells with significant cell death only being observed past 24 h (Fig. 7a and b). Importantly, cell viability was recovered upon reconstitution of WT G6PD into G6PD KO cells (Fig. 7c). This form of cell death was ROS dependent, as antioxidant NAC was able to reduce p-JNK level and protect against cell death induced by O/A treatment in G6PD KO cells (Supplementary Figs. S3e, S7a, and S7b). Interestingly, NAC was not able to recover PINK1 or p-Ub levels (Supplementary Fig. S7c and d) or degradation of mitochondrial proteins (Fig. 3g and h), suggesting again that ROS are not directly implicated in PINK1 stabilization and activation and eventually mitophagy in G6PD KO cells upon O/A treatment. Furthermore, the NADP^+^/NADPH ratio increased in G6PD KO cells after O/A treatment, and NAC treatment did not lower this ratio to control levels (Supplementary Fig. S7e), indicating the inability of G6PD KO cells to regulate the NADP^+^/NADPH ratio during O/A treatment even in the presence of NAC.

Recently, we showed that Spautin-1, an inhibitor of ubiquitin-specific peptidase 10 (USP10) and USP13, stabilizes PINK1 by binding to OMM proteins TOM70 and TOM20, preventing PINK1 import and degradation by PARL [61]. Treatment of G6PD KO cells with Spautin-1 partially recovered mitochondrial protein degradation and Ub phosphorylation (Fig. 7d and e; Supplementary Fig. S7f), consistent with the notion that G6PD deletion triggers PINK1 destabilization and hence mitophagy inhibition. Importantly, this recovery in mitophagy corresponded to a partial recovery in cell viability in Spautin-1- and O/A-treated G6PD KO cells (Fig. 7f). Therefore, the mitophagy impairment in G6PD KO cells contributes, at least partially, to the reduced cell viability upon O/A treatment. Taken together, mitophagy regulation by G6PD serves a physiological function to maintain cell viability under mitochondrial stress caused by O/A treatment.

## Discussion

In this study, we have identified a novel role of G6PD in PINK1-Parkin-mediated mitophagy. First, through a whole-genome CRISPR-Cas9 screening, *G6PD* was revealed to be one of the top genes influencing PINK1-Parkin-mediated mitophagy. Next, we validated the role of G6PD in mitophagy by using both genetic deletion and pharmacological inhibition of G6PD. The positive role of G6PD in mitophagy is related to the catalytic activity of G6PD, but it does not depend on the PPP. Mechanistically, G6PD localizes onto the OMM where it promotes PINK1 stability via direct protein-protein interaction. Finally, G6PD deficiency sensitizes cells to O/A-induced cell death, a process related to ROS accumulation and oxidative stress. In summary, we reveal a novel function of G6PD in PINK1-Parkin-mediated mitophagy through regulating PINK1 stability and activity (Fig. 7g).

G6PD is well-characterized as the rate-limiting enzyme of the PPP, in which G6PD catalyzes the conversion of G6P to 6-phosphogluconolactone and NADP^+^ to NADPH. Our investigation into the role of G6PD in mitophagy regulation revealed that the PPP was not involved in the mitophagy inhibition caused by G6PD deletion (Fig. 3; Supplementary Fig. S3). However, the lack of catalytic activity hindered the ability of G6PD to regulate mitophagy, while catalytically activating G6PD enhanced mitophagy. PGLS KO, which would cause an accumulation of 6-phosphogluconolactone due to the inability to be converted to 6-phosphogluconate, did not cause an increase in mitophagy activation. Thus, it is unlikely that the glycolysis side-products are involved in mitophagy. On the other hand, while the conversion of G6P to 6-phosphogluconolactone is specific to glucose metabolism, the conversion of NADP^+^ to NADPH is not. Taken together, this suggests that while the PPP *per se* is not involved, there is a possible role of the NADP^+^/NADPH ratio in mitophagy regulation by G6PD.

The maintenance of the NADP^+^/NADPH ratio by G6PD is vital toward healthy redox homeostasis as NADPH is involved in the conversion of GSSG to GSH to act as a reducing factor in antioxidant defense [49]. This is particularly important in the context of the mitochondria, which is the central organelle for ROS production [62–68]. Indeed, many previous studies have shown that G6PD regulates mitochondrial dysfunction caused by oxidative stress [58, 69]. G6PD-deficient cells have been shown to exhibit high ROS levels and mitochondria-dependent apoptosis [70]. However, while the lack of G6PD caused increased ROS-dependent cell death in O/A-treated G6PD KO cells, we found that the mitophagy inhibition was not reversed by treatment with the antioxidant NAC, suggesting that it is not caused by ROS accumulation (Fig. 7). Importantly, the increase in the NADP^+^/NADPH ratio in O/A-treated G6PD KO cells was not reversed by NAC treatment either (Supplementary Fig. S7e), pointing towards a possible link between the perturbation of the NADP^+^/NADPH ratio and the inhibition of mitophagy. Moreover, Spautin-1 treatment resulted in a recovery of mitophagy phenotypes as well as cell viability in O/A-treated G6PD KO cells (Fig. 7d−f; Supplementary Fig. S7f), thus providing a potential mechanism behind PINK1 regulation by G6PD as well as its contribution toward cellular health during mitochondrial stress conditions. Overall, our findings highlight an important novel role for the catalytic activity of G6PD in mitophagy that is uncoupled from its known roles in the PPP and redox homeostasis.

G6PD has been consistently reported to localize at the mitochondria [57–59, 71]. However, any reported effect of G6PD on the mitochondria is known to be indirect, mostly by the regulation of mitochondrial ROS through the PPP to prevent apoptosis [70, 72]. Consistent with previous studies, we found that G6PD localized at the mitochondria (Fig. 6a–c). Strikingly, we showed that G6PD interacted with PINK1 (Fig. 6d–f). Interestingly, while PINK1 was stabilized during O/A treatment, the mitochondrial localization of G6PD did not change. Nevertheless, this interaction appeared to be relevant to the activity of PINK1 and thus the level of mitophagy and involved the C-terminal region of G6PD (Supplementary Fig. S6). Specifically, there was an inhibition of PINK1 stabilization in O/A-treated G6PD KO cells, and consequently a lack of ubiquitin phosphorylation and amplification of the mitophagy signal (Fig. 5). Thus, our work demonstrates that G6PD not only acts indirectly on mitophagy and mitochondrial health through maintaining low ROS levels, but also acts directly with the mitophagy machinery by interacting with PINK1.

As mitochondria are central organelles for energy production and homeostasis [73, 74], and mitophagy is an intricate, multi-stage process, PINK1-Parkin-mediated mitophagy is understandably regulated at multiple steps. For example, PINK1 is comprehensively regulated from the gene level to the protein level. At the transcription level, *PINK1* may be positively regulated by nuclear respiratory factor-1 (*NRF-1*), a gene essential for mitochondrial biogenesis, or nuclear factor-κB (NFκB), an oxidative stress-activated transcription factor [75, 76]. On the other hand, transcription of *PINK1* may be downregulated by nuclear p53 [77]. Outside of the nucleus, PINK1 is generally regulated with regards to mitochondrial import [16]. To date, PINK1 has not been reported to be affected or regulated by NADP^+^ or NADPH. It remains to be discovered how PINK1 dynamics may be affected by the NADP^+^/NADPH ratio, and further studies are required to characterize the G6PD-PINK1 interaction and its significance to mitophagy.

### Limitations of the study

There are several limitations of this study. First, our results mainly worked in the HeLa 3+ model. It would be important to recapitulate these findings in other cell lines to further elucidate the relationship between G6PD and mitophagy. Second, as G6PD mainly exists in the cytosol, the mechanism underlying its mitochondrial localization remains to be further elucidated. Third, we do not have any evidence from disease models *in vivo* or human specimens showing the link between G6PD and mitophagy.

In conclusion, our results demonstrate that G6PD regulates PINK1 stabilization during mitophagy via interaction at mitochondria (Fig. 7g). Because the catalytic activity of G6PD is required to some extent, PINK1 may work downstream of G6PD, a process partly depending on the NADP^+^/NADPH ratio that is maintained by G6PD. This new function of G6PD in the regulation of mitophagy thus connects several important cellular processes, namely glucose metabolism, redox homeostasis, and mitochondrial quality control.

## Materials and methods

### Chemicals

2-Deoxy-d-glucose (2-DG) (D6134), Antimycin A (A8674), Bay-11-7082 (B5556), N-Acetyl-L-cysteine (NAC) (A9165), Oligomycin (15351), Parthenolide (P0667), and Wedelolactone (W4016) were purchased from Sigma-Aldrich. 6-AN (10009315), 7-aminoactinomycin D (7-AAD) (11397), DHEA (15728), MG132 (10012628), and Q-VD-OPH (15260) were purchased from Cayman Chemical. AG1 (HY-123962) and Spautin-1 (HY-12990) were purchased from MedChemExpress.

### Cell lines and cell culture

HeLa and HEK293T cell lines were purchased from the American Type Culture Collection cell bank. HeLa-YFP-Parkin cell line was a gift from Professor Richard Youle. All cell lines were maintained in Dulbecco’s modified Eagle’s medium (DMEM, HyClone, SH30022.01) with 10% fetal bovine serum (Hyclone, SV30160.03) in a 5% CO_2_ incubator at 37°C. Cultured cells were passaged once they reached 80%–90% confluency. For drug treatment, passaged cells were cultured overnight in a normal medium before the addition of the indicated drug concentration for the duration as described in the figure legends for each experiment. A standard concentration of O/A (5 µmol/L and 1 µmol/L, respectively) was used for all treatments.

### Plasmids, siRNA, and transfection

All G6PD mutants were generated using WT G6PD plasmid (Addgene, #41521) as a template. The primers used to generate the mutants are summarized in Supplementary Table S1. Transfection of G6PD or G6PD mutants was performed directly into cells while seeding to a confluency of 50%–70% using Lipofectamine 3000 (Invitrogen, L3000075) according to the manufacturer’s instructions. After transfection for 24 h, the indicated treatment was performed and cells were subsequently subjected to live-cell imaging, western blotting, and immunoprecipitation, as detailed below.

For CRISPR KO cell lines, sgRNAs were obtained from Integrated DNA Technologies. The sgRNAs targeting indicated genes were transfected into cells while seeding to a confluency of 20%–30% using Lipofectamine RNAiMAX according to the manufacturer’s instructions. Cells were maintained when they reached confluency and subjected to western blotting analysis to verify the presence of gene knockout. The sgRNA sequences are summarized in Supplementary Table S2.

### Generation of HeLa 3+ cells

HeLa 3+ cells co-expressing mCherry-Parkin, mito-GFP, and miRFP-FLAG-Cas9 were established in a multi-step process. Firstly, plasmids encoding retroviral vector components VSV-G envelope (pMD2.G, Addgene #12259) and Gag-Pol (pUMVC-gagpol, Addgene #8449), along with the plasmid encoding mCherry-Parkin (Addgene #59419), were co-expressed in HEK293T cells. After overnight incubation, the supernatant was harvested and replaced with fresh DMEM. After another overnight incubation, the supernatant was harvested again and pooled with the first collection. The pooled supernatant was filtered using a 0.22-μm filter unit before being added to wild-type HeLa cells (target cells) in the presence of 8 µg/mL polybrene. Subsequently, these HeLa cells were incubated with the retrovirus-containing media for another two nights before the selection was done by fluorescence-activated cell sorting (FACS, excitation wavelength 540−590 nm; emission wavelength: 550−650 nm). The resultant cells were mCherry-Parkin positive HeLa cells (HeLa-mCherry-Parkin).

Next, mito-GFP and miRFP-FLAG-Cas9 were expressed sequentially using lentiviral vectors. Plasmids encoding vector components VSV-G envelope (pMD2.G), Gag-Pol (pMDLg/pRRE, Addgene #12251), Rev (pRSV-Rev, Addgene #12253), along with the plasmid encoding mito-GFP (pLV-mitoGFP, Addgene #44385), were co-expressed in HEK293T cells. Incubation and harvesting were conducted as described above, with HeLa-mCherry-Parkin cells serving as the target cells. The selection was done by FACS with excitation/emission wavelength of 488/509 nm and the resultant cells were mCherry-Parkin and mito-GFP positive HeLa cells (HeLa-mCherry-Parkin-mito-GFP).

Lastly, miRFP-FLAG-Cas9 (pFU-Cas9-T2a-miRFP670, gift from Professor Naiyang Fu) was expressed in the target cells, HeLa-mCherry-Parkin-mito-GFP cells, similar to the lentiviral expression of mito-GFP as described above. The selection was done by FACS with excitation/emission wavelength of 674/703 nm. These cells, with stable expression of the three fluorescent proteins, were named as HeLa 3+ cells.

The parental population of HeLa 3+ cells was subjected to single-cell selection to ensure uniform expression of the overexpressed proteins. Two clones, #12 and #31, were selected to undergo the screening process.

### Genome-wide CRISPR/Cas9 knockout screen

The Human Two Plasmid Activity-Optimized CRISPR Knockout Library constructed by David Sabatini/Eric Lander and colleagues was obtained from Addgene (#1000000095) [41]. The lentivirus preparation was conducted as described previously with some modifications [78]. Briefly, the lentivirus of the library was prepared by virus packaging in HEK293 cells. For the screening process, cells were seeded and cultured in T150 flasks. About 300 million cells in total were incubated with a medium containing lentivirus of the library and 2 μg/mL polybrene at a multiplicity of infection (MOI) = 0.3–0.5 (~70%–50% cell death induced by puromycin, respectively) for two days. After infection, the cells were selected with 10 μg/mL puromycin in fresh media for two days and then cultured in fresh medium without puromycin for a few more days. Surviving cells expressing the sgRNAs were treated with O/A (5 μmol/L and 1 μmol/L, respectively) for 42 h. Cells were trypsinized and dyed with 7-AAD (Cayman Chemicals) to exclude dead cells. Cells were then subjected to FACS using GFP as the readout (excitation/emission wavelength: 488/509 nm). Cells falling into the top 8% and bottom 8% of GFP fluorescence intensity were collected for further analysis. Cells in the top 8% of GFP fluorescence intensity would represent cells exhibiting an inhibition of mitophagy, indicating that these cells express sgRNAs which have eliminated positive regulators of mitophagy. Cells in the bottom 8% would serve as a baseline control.

The genomic DNA was extracted from both the top 8% and bottom 8% of GFP-expressing cells according to the manufacturer’s instructions (Bio Basic, One-4-All Genomic DNA Miniprep Kit). NGS libraries were prepared with specific barcode primers by PCR (Supplementary Table S3). Multiplex NGS libraries were sent to two sequencing vendors, Genome Institute of Singapore and Macrogen, to identify the sgRNAs in each sample. Normalization, sgRNA modeling, and ranking were done on the MAGeCK algorithm as described previously [78, 79].

Alignment to the sgRNA library was subsequently carried out before the calculation of the abundance of each sgRNA. The log_2_ fold change in abundance of each sgRNA was calculated for the top 8% of GFP-expressing cells using the sgRNA counts in the bottom 8% of GFP-expressing cells as the control. Gene-based CRISPR scores (CS) were defined as the 5th-highest log_2_ fold change of all sgRNAs targeting a given gene or the average log_2_ fold change of all sgRNAs targeting a given gene.

### Live cell imaging

Cells stably expressing fluorescent proteins were seeded in a 35-mm glass-bottom dish to a final confluency of 80%. For transfected cells, transfection was done during seeding to a confluency of 40%. All cells were incubated overnight. After the indicated treatment, cells were subjected to imaging with a Leica fluorescence microscope at indicated time points.

### SDS-PAGE and western blotting analysis

Cells were washed in phosphate-buffered saline (PBS) before lysis in SDS lysis buffer (containing 62.5 mmol/L Tris pH 6.8, 2% SDS, 1 mmol/L EDTA, 20% glycerol, 2 mmol/L dithiothreitol, and 1× Halt™ Protease and Phosphatase Inhibitor Cocktail), and subsequent boiling for 10 min. The DC Protein Assay (Bio-Rad, #5000112) was used to estimate the protein concentration. After which, the sample was diluted with 4× Laemmli sample buffer (Bio-Rad, #1610747), resolved by SDS-PAGE, and then transferred onto PVDF membranes (Bio-Rad, #1620177). Blocking of the membranes with 5% nonfat milk was performed and membranes were incubated overnight at 4°C with the indicated primary antibodies in the key resources table. Membranes were then incubated at room temperature with HRP-conjugated secondary antibodies for 1 h. Subsequent detection of chemiluminescence signals was performed using ImageQuant LAS500 (GE Healthcare) with Immobilon Forte Western HRP substrate (Merck-Millipore, WBLUF0500) or Amersham ECL Select Western Blotting Detection Reagent (GE Healthcare, RPN2235). The following primary antibodies were used: Cell Signaling Technology: AMPKα (#5832), GFP (#2956), SAPK/JNK (#9252), K48-linkage Specific Polyubiquitin (#8081), Mitofusin-1 (#14739), Mitofusin-2 (#11925), phospho-AMPKα (Thr172) (#2535), phospho-SAPK/JNK (Thr183/Tyr185) (#4668), phospho-Ubiquitin (Ser65) (#62802), Parkin (#4211), PINK1 (#6946), and TOM20 (#42406); Santa Cruz Biotechnology: G6PD (sc-373886) and HA tag (sc-7392); Sigma-Aldrich: FLAG® M2 (F1804), α-Tubulin (T6199), and β-Actin (A5441); Abcam: GAPDH (ab8245), mCherry (ab167453), and MTCO2 (COXII) (ab110258); BD Transduction Laboratories: TIM23 (611222); Proteintech: VDAC1/2 (10866-1-AP). The secondary antibodies used were Cell Signaling Technology: Anti-mouse IgG, HRP-linked (#7076) and anti-rabbit IgG, HRP-linked (#7074).

All primary antibodies were diluted according to protocols in 5% (w/v) bovine serum albumin in Tris-buffered saline with 0.1% (v/v) Tween-20 (TBST) containing 0.1% (w/v) sodium azide. The concentration of secondary HRP-linked antibodies used was 1:2000 diluted in TBST with 5% (w/v) nonfat milk.

### Immunoprecipitation

HeLa 3+ cells were transfected with myc-PINK1 or myc-tagged G6PD truncation mutants. Twenty-four hours after transfection, cells were treated as indicated. Subsequently, cells were washed with cold PBS and lysed in 800 μL IP lysis buffer (25 mmol/L Tris hydrochloric acid pH 7.4, 150 mmol/L NaCl, 1% NP-40, 1 mmol/L EDTA, and 5% glycerol) with 1× Halt™ Protease and Phosphatase Inhibitor Cocktail on ice for 45 min, with periodic mixing. The lysates were then subjected to sonication and centrifuged at 12,000 *g* for 15 min at 4°C. Protein concentration was determined using DC Protein Assay and a portion of the supernatants were aliquoted as the input. The remaining supernatants were pre-cleared with Pierce™ Protein A/G Agarose beads (Thermo Fisher, 20421) for 2 h at 4°C and centrifuged at 1,000 *g* for 2 min at 4°C to remove the beads. The supernatants were further mixed with 20 μL agarose-conjugated G6PD (Santa Cruz Biotechnology, sc-373886 AC), 20 μL agarose-conjugated PINK1 antibody (Santa Cruz Biotechnology, sc-518052 AC), 20 μL ANTI-FLAG® M2 Affinity Gel (Merck Millipore, A2220), 5 μL anti-Myc-tag antibody (MBL Life Science, M192-3), or control IgG overnight at 4°C. For free antibody IP, Protein A/G beads were added and allowed to conjugate for 2 h. Subsequently, beads were washed three times with 1 mL IP lysis buffer before boiling for 10 min with 25 μL 2× Laemmli sample buffer (Bio-Rad, #1610737) and kept for western blotting analysis. The following isotype control antibody was used: Cell Signaling Technologies: Mouse (G3A1) monoclonal IgG1 Isotype Control (#5415).

### Protein–protein interaction assay

FLAG-G6PD was purified by pull-down with FLAG M2 monoclonal antibody affinity gel (Sigma-Aldrich, A2220). After treatment, HeLa mCherry-Parkin cells transfected with FLAG-G6PD were washed with cold PBS and lysed in 1 mL lysis buffer (25 mmol/L Tris hydrochloric acid pH 7.4, 150 mmol/L NaCl, 1% NP-40, 1 mmol/L EDTA, and 5% glycerol) with 1× Halt™ Protease and Phosphatase Inhibitor Cocktail on ice. The lysates were then subjected to sonication and centrifuged at 12,000 *g* for 15 min at 4°C. The supernatants were pre-cleared with 10 μL mouse IgG (Santa Cruz Biotechnologies, sc-2343) overnight at 4°C followed by incubation with Pierce™ Protein A/G Agarose beads for 3 h. Samples were centrifuged at 17,000 *g* for 15 min at 4°C to remove the beads. Further mixing of the supernatants with 10 μL FLAG M2 monoclonal antibody affinity gel (Sigma-Aldrich, A2220) was carried out for 3 h at 4°C. Subsequently, beads were washed thrice with 1 mL lysis buffer. The purified protein on the beads was incubated with 100 ng recombinant human PINK1 protein (Novus Biologicals, NBP1-72481) in 1 mL lysis buffer for 2 h at room temperature. After incubation, the beads were washed thrice with 1 mL lysis buffer and subjected to western blotting analysis.

### Mitochondrial fractionation

Mitochondria were isolated from cells as previously described [80] with minor modifications. Briefly, cells (two 10-cm dishes or one 15-cm dish) were collected and homogenized using a Dounce homogenizer (Wheaton, #357538) for 50 strokes on ice in 700 μL separation buffer (250 mmol/L sucrose, 5 mmol/L MgCl_2_, 50 mmol/L Tris hydrochloric acid pH 7.4, and 1× Halt™ Protease and Phosphatase Inhibitor Cocktail). Subsequently, samples were centrifuged twice at 800 *g* for 10 min at 4°C to obtain crude supernatants, before further centrifugation at 11,000 *g* for 15 min at 4°C to harvest crude mitochondrial pellets. The pellets were resuspended thrice in 800 μL separation buffer and centrifuged at 11,000 *g* for 10 min at 4°C. In the final step, SDS lysis buffer was used to lyse the centrifuged mitochondria for western blotting analysis.

### PLA

YFP-Parkin HeLa cells were seeded on 18-mm cover slips to 70%–80% confluency. Cells were treated with O/A (5 μmol/L and 1 μmol/L, respectively) for 90 min. Afterward, cells were washed with PBS and fixed with 100% methanol for 15 min at 4°C. After fixation, cells were permeabilized with 0.1% NP-40 for 5 min. The PLA was performed using Duolink® *In Situ* Detection Reagents Red (DUO92008, Sigma-Aldrich) together with Duolink® *In Situ* PLA® Probe Anti-Rabbit PLUS (DUO92002) and Anti-Mouse MINUS (DUO92004) according to the manufacturer’s instructions. Briefly, after permeabilization, the samples were blocked and then incubated overnight with antibodies against G6PD or myc and TOM20 or PINK1. Samples were then incubated with the MINUS and PLUS PLA probes corresponding to the primary antibodies used, followed by ligation and amplification in the presence of Texas-Red labeled oligos to generate the PLA signal. All incubations were performed in a 37°C humidity chamber using a volume of 40 μL per sample. After incubation, cells were labeled with 1 μg/mL DAPI in PBS. Finally, the samples were mounted on a glass slide with ProLong® Diamond Antifade Mountant mounting medium. Samples were observed on Zeiss LSM 700 inverted confocal microscope (Carl Zeiss) with four laser lines (405/488/555/639 nm) using a 63×/1.15 oil immersion objective lens. Zeiss Zen software was used to acquire images from an AxioCam MRm monochromatic CCD camera.

### Measurement of NADP^+^/NADPH ratio

The concentration of NADPH was determined by the NADP/NADPH Quantification Colorimetric Kit (Sigma Adrich, MAK038) following the manufacturer’s instructions. Briefly, cells from a six-well plate (80% confluency) were washed with cold PBS and subjected to trypsinization. Cells were lysed in 500 μL extraction buffer on ice and centrifuged at 10,000 *g* for 10 min at 4°C. A small portion of the supernatant was aliquoted to determine the protein concentration and the rest was subjected to filtration through a 10 kDa Molecular Weight Cut-Off filter (Merck Millipore, MRCPRT010) for deproteinization. A portion of the deproteinized sample was heated at 60°C for 30 min to denature NADP^+^ and the rest was used to measure total NADPH. Samples were then subjected to the NADPH assay to establish the amount and ratio of total NADP^+^/NADPH. Absorbance was measured at 450 nm.

### Measurement of G6PD activity

G6PD activity in HeLa 3+ cells was determined by measuring the production rate of reduced nicotinamide adenine dinucleotide (NADH) at 450 nm using a glucose-6-phosphate dehydrogenase assay kit (Abcam, ab102529) according to the manufacturer’s instructions. Briefly, cells were harvested in cold PBS and homogenized by pipetting. Samples were centrifuged at 12,000 *g* for 5 min at 4°C to remove insoluble material. Afterwards, the reaction mix was added to each sample in a 96-well plate. The plate was measured at 450 nm in a microplate reader in kinetic mode up to 60 min with readings recorded at intervals. Fold change of kinetic activity was calculated using the changes in the optical density (OD) at 450 nm.

### Measurement of cell viability

Cell viability was analyzed via Trypan Blue exclusion test. Cells were trypsinized and diluted in a 1:1 ratio with 0.4% Trypan Blue Solution (Thermo Fisher, 15250061). The solution was loaded into a hemocytometer and counted under a light microscope at 20× magnification. The absolute numbers of live cells (white) and dead cells (blue) were counted. At least 500 total cells per sample per repeat were counted. % viable cells = [number of live cells ÷ (number of live cells + number of dead cells)] × 100%.

### Statistical analysis

All imaging and immunoblotting data presented were representatives from at least three independent repeated experiments. All numeric data were presented as mean ± SD from at least three independent experiments. The *P* value was analyzed by Student’s *t*-test. The following denote the *P* value for statistical significance: ^*^P < 0.05; ^**^P < 0.01; ^***^P < 0.001.

## Supplementary Material

loae040_suppl_Supplementary_Figures_S1-S7

loae040_suppl_Supplementary_Tables_S1-S3

## Data Availability

All data relevant to the study are included in the article or uploaded as supplementary information. The data used to support the findings of this study will be available on request.
