# Supplementary material for: Glucose-6-phosphate dehydrogenase regulates mitophagy by maintaining PINK1 stability
Source: Life Metab. 2024 Dec 13;4(1):loae040. doi: 10.1093/lifemeta/loae040 (PMC11749863; doi:10.1093/lifemeta/loae040)
Supplement: loae040_suppl_Supplementary_Figures_S1-S7 [file loae040_suppl_supplementary_figures_s1-s7.pdf]

Supplementary Figure S1 Main hits and pathways identified by the whole-genome screening, related to Fig. 1. (a) Schematic diagram of the process identifying top hits from the whole-genome CRISPR-Cas9 screen. HeLa 3+ cells containing the sgRNA library were treated with O/A (5 μmol/L and 1 μmol/L, respectively) for 42 h to ensure depletion of mito-GFP. Cells were then subjected to fluorescence-activated cell sorting (FACS) using GFP as a readout. The top 8% of GFP-expressing cells were collected as the target pool and the bottom 8% was collected as the control pool. Subsequently, DNA extraction and amplification were conducted on the samples. Samples were sent for next-generation sequencing (NGS). Partially created with BioRender.com. (b) Criteria for the hits and number of genes in each dataset. Datasets were produced from two individual clones (#12 and #31) that were each sent to two different vendors (Genome Institute of Singapore (GIS), and Macrogen) for sequencing. (c) Overlap analysis of the chosen genes. Datasets #2 and #4, both from clone #31, were compared. The overlap consisted of 263 genes. (d) Pathway analysis of the hits falling within the analysis criteria using Reactome. (e) The criteria identifying G6PD as a top hit in the datasets.

Supplementary Figure S2 G6PD KO cells created using an independent clone and sgRNA exhibit mitophagy inhibition, related to Fig. 2. (a) Immunoblotting analysis for verification of G6PD knockout in clone #31 cells using two different sgRNAs. (b) Quantification of mCherry-Parkin translocation to the mitochondria by co-localization with mito-GFP as seen in Fig. 2b. A total of at least 100 cells from three independent experiments were counted. (c) Live cell images showing Parkin translocation in G6PD KO HeLa 3+ (clone #31) cells created with G6PD sgRNA #2. Cells were treated with O/A (5 μmol/L and 1 μmol/L, respectively) for 4 h. (Left) Live images taken with a Leica fluorescence microscope. Scale bar: 20 μm. (Right) Quantification of mCherry-Parkin translocation to the mitochondria by co-localization with mito-GFP as seen in the left panel. A total of at least 100 cells from three independent experiments were counted. (d) WT and G6PD KO HeLa 3+ cells (clone #31) created with sgRNA #2 were treated with O/A (5 μmol/L and 1 μmol/L, respectively) for indicated time points and subjected to immunoblotting analysis. (e) WT and G6PD KO HeLa 3+ cells (clone #12) created using both sgRNA #1 and #2 were treated with O/A (5 μmol/L and 1 μmol/L, respectively) for 4 h. (top) Live images were taken with a Leica fluorescence microscope. Scale bar: 20 μm. (bottom) Quantification of mCherry-Parkin translocation to the mitochondria by co-localization with mito-GFP as seen in the top panel. A total of at least 100 cells from three independent experiments were counted. (f) WT and G6PD KO HeLa 3+ cells (clone #31) created with sgRNA #1 and #2 were treated with CCCP (10 μmol/L) for indicated time points and subjected to immunoblotting analysis. (g) Quantification of mCherry-Parkin translocation to the mitochondria by co-localization with mito-GFP as seen in Fig. 2e. A total of at least 100 cells from three independent experiments were counted. (h) G6PD activity assay for G6PD KO and reconstituted cells. G6PD KO HeLa 3+ cells were transfected with WT G6PD for 24 h. Cells were collected and subjected to G6PD activity assay (colorimetric). A kinetic curve of G6PD activity is shown. RE: reconstituted; *x*-axis: minutes; *y*-axis: fold change of absorbance at 450 nm, indicating activity. Quantification data in (b), (c), (e), (g), and (h) are presented as mean ± SD of three independent experiments. ns: not significant. $^{*}P < 0.05$; $^{**}P < 0.01$; $^{***}P < 0.001$.

Supplementary Figure S3 The effects of PPP perturbation on mitophagy, related to Fig. 3.

(a) Quantification of mCherry-Parkin translocation to the mitochondria by co-localization with mito-GFP as seen in Fig. 3a. A total of at least 100 cells from three independent experiments were counted. (b) Immunoblotting analysis of glucose starvation markers and mitochondrial protein levels. HeLa 3+ cells were treated with glucose starvation media (GS) or glucose starvation media supplemented with 5 mmol/L 2-deoxyglucose (GS + 2-DG) for 2 h. Cells were then treated with O/A (5 µmol/L and 1 µmol/L, respectively) for 4 h and subjected to immunoblotting analysis. Loading control is shared with Fig.e 3b. (c) Quantification of mCherry-Parkin translocation to the mitochondria by co-localization with mito-GFP as seen in Fig. 3c. A total of at least 100 cells from three independent experiments were counted. (d) Quantification of mCherry-Parkin translocation to the mitochondria by co-localization with mito-GFP as seen in Fig. 3f. A total of at least 100 cells from three independent experiments were counted. (e) (top) Immunoblotting analysis of p-JNK levels as a measurement of ROS. HeLa 3+ cells (WT and G6PD KO) were treated with NAC (10 mmol/L) and O/A (5 µmol/L and 1 µmol/L, respectively) for 4 h. (bottom) Quantification of p-JNK levels in the top panel, normalized to total JNK. Fold change was calculated using wild-type, untreated cells as the baseline. Quantification data in (a), (c), (d), and (e) are presented as mean ± SD of three independent experiments. ns: not significant. $^{*}P < 0.05$; $^{***}P < 0.001$.

Supplementary Figure S4 The effect of catalytic inhibition of G6PD on Parkin translocation, related to Fig. 4. (a) Quantification of mCherry-Parkin translocation to the mitochondria by co-localization with mito-GFP as seen in Fig. 4a. A total of at least 100 cells from three independent experiments were counted. (b) Quantification of mCherry-Parkin translocation to the mitochondria by co-localization with mito-GFP as seen in Fig. 4c. A total of at least 100 cells from three independent experiments were counted. (c) Changes in mCherry-Parkin translocation measured by live cell imaging. G6PD KO HeLa 3+ cells were transfected with WT G6PD or G6PD BN variant for 24 h. Cells were treated with O/A (5 µmol/L and 1 µmol/L, respectively) for 2 h. (top) Live images were taken with a Leica fluorescence microscope. Scale bar: 20 µm. (bottom) Quantification of mCherry-Parkin translocation to the mitochondria by co-localization with mito-GFP as seen in the top panel. A total of at least 100 cells from three independent experiments were counted. Quantification data are presented as mean ± SD of three independent experiments. BN, Bangkok noi. ns: not significant. $^{*}P < 0.05$; $^{**}P < 0.01$; $^{***}P < 0.001$.

Supplementary Figure S5 G6PD KO cells show a decrease in PINK1 and p-Ub levels upon mitophagy induction, related to Fig. 5. (a) Quantification of p-Ub (top) and PINK1 (bottom) levels in lysates after 4 h of O/A treatment as seen in Fig. 5a. Fold change was calculated using wild-type, untreated cells as the baseline. (b) Detection of p-Ub and PINK1 protein levels by immunoblotting analysis. WT and G6PD KO HeLa 3+ cells (clone #31) created with sgRNA #1 and #2 were treated with CCCP (10 µmol/L) for indicated time points and subjected to immunoblotting analysis. Loading control and G6PD blot are shared with Supplementary Fig. S2f. (c) Quantification of p-Ub (top) and PINK1 (bottom) levels in lysates after 4 h of O/A treatment as seen in Fig. 5c. Fold change was calculated using wild-type, untreated cells as the baseline. (d) Detection of mitochondrial protein levels by immunoblotting analysis. G6PD KO HeLa 3+ cells were transfected with WT G6PD or G6PD BN for 24 h. Cells were then treated with O/A (5 µmol/L and 1 µmol/L, respectively) for 4 h and lysates were subjected to immunoblotting analysis. BN:

Bangkok noi. (e) Quantification of p-Ub (top) and PINK1 (bottom) levels in O/A-treated cells as seen in Fig. 5f. Fold change was calculated using wild-type, untreated cells as the baseline. (f) Quantification of mCherry-Parkin translocation to the mitochondria by co-localization with mito-GFP as seen in Fig. 5g. A total of at least 100 cells from three independent experiments were counted. (g) Quantification of cleaved PINK1 levels in lysates treated with both MG132 and O/A as seen in Fig. 5j. Fold change was calculated using wild-type, untreated cells as the baseline. Data in (a), (c), (e), (f), and (g) are presented as mean ± SD of three independent experiments. ns: not significant. $^{*}P < 0.05$; $^{**}P < 0.01$; $^{***}P < 0.001$.

Supplementary Figure S6 The C-terminal domain of G6PD is important for binding to PINK1, related to Fig. 6. (a) Schematic diagram of the three different truncated myc-tagged G6PD mutants. G6PD contains a $NADP^+$-binding domain at its N terminal and a C-terminal domain containing a dimer interface for the formation of the G6PD dimer. (b) PLA performed on YFP-Parkin HeLa cells overexpressing truncated G6PD mutants. Cells were transfected with the indicated mutants for 24 h before treatment with O/A (5 µmol/L and 1 µmol/L, respectively) for 90 min. Assay was performed using myc and PINK1 primary antibodies. Red, PLA signal; grey (pseudocoloured), YFP-Parkin; blue, DAPI. Scale bar: 20 µm. (c) Detection of p-Ub and PINK1 protein levels by immunoblotting analysis. YFP-Parkin HeLa cells were treated with O/A (5 µmol/L and 1 µmol/L, respectively) for 90 min. Cell lysates were subjected to immunoblotting analysis with the indicated antibodies. EV: empty vector (myc); FL: full-length.

Supplementary Figure S7 Mitophagy inhibition in G6PD KO cells is not due to ROS, but may be related to the $NADP^+$/NADPH ratio, related to Fig. 3 and Fig. 7. (a) Detection of cell viability using bright field imaging. WT and G6PD KO HeLa 3+ cells were treated with NAC (10 mmol/L) and O/A (5 µmol/L and 1 µmol/L, respectively) for 6 h. Cells were subjected to bright field imaging. Scale bar: 200 µm. (b) Quantification of the data from (a). (c) Mitochondrial protein levels detected by immunoblotting analysis. WT and G6PD KO HeLa 3+ cells were treated with NAC (10 mmol/L) and O/A (5 µmol/L and 1 µmol/L, respectively) for 4 h. Lysates were immunoblotted with the indicated antibodies. (d) Quantification of the data from (c). Fold change was calculated using wild-type, untreated cells as the baseline. (e) Measurement of the $NADP^+$/NADPH ratio. HeLa 3+ cells (WT and G6PD KO) were treated with NAC (10 mmol/L) and O/A (5 µmol/L and 1 µmol/L, respectively) for 4 h. Cells were subjected to an $NADP^+$/NADPH quantitation assay. (f) Quantification of MFN1 levels in cells treated with Spautin-1 and O/A as seen in Fig. 7d. Fold change was calculated using wild-type, untreated cells as the baseline. Data in (b), (d), (e), and (f) are presented as mean ± SD of three independent experiments. ns: not significant. $^{*}P < 0.05$; $^{**}P < 0.01$; $^{***}P < 0.001$.

Supplementary Figure S1

a

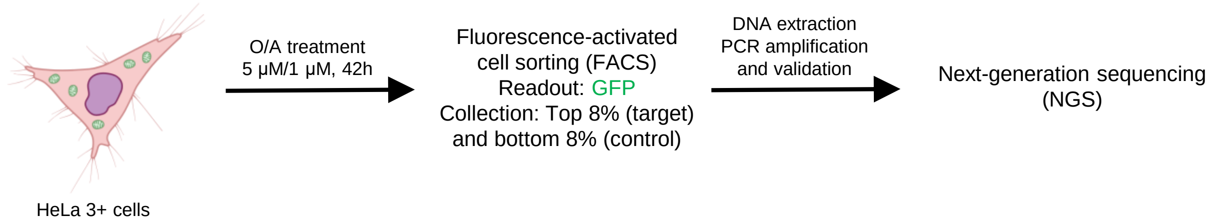

b

Dataset	Clone	Sequencing vendor	No. of genes	Criteria
#1	12	GIS	897	$p < 0.05$<br>> 3 sgRNAs with fold enrichment > 2
#2	31	GIS	862	
#3	12	Macrogen	912	
#4	31	Macrogen	1077	

c

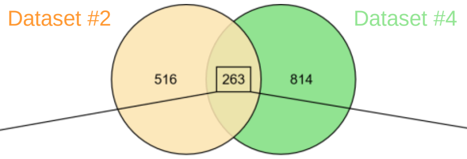

PINK1	PGAM1	NOL6	VARS	SRFBP1	RIOK2	DDX21	PGK1	NUDC	UBA52	PPP1R8	PPP2R1A	GTPBP4	INTS3	AMY1A	GTF3C1	SGIP1	DIMT1	NMD3
PARK2	DAXX	LAS1L	CIRH1A	ACTR10	TAF1B	RPS25	EIF1AX	TBCD	NIP7	YTHDF2	NAA10	MTOR	ZSCAN10	DHX15	WDR7	MYBBP1A	CPNE6	EXOSC4
**G6PD**	EPRS	MTPAP	LTV1	OSGEP	ZCCHC9	WDR74	ADAM30	NVL	GPN2	EIF4E	ZMAT5	TMEM199	VPS52	GLCCI1	BANF1	C16orf80	NUDT16L1	CCT7
PIAS1	CARS	GPI	EXOSC10	CCNH	GMPPB	NHP2	FTSJ3	CDS2	CENPW	KARS	AK6	CHP2	BIRC2	ITPRIPL1	TAF7	MPHOSPH10	LITAF	CCDC64B
ATRX	NARS	TTC27	SARS	PWP2	PES1	ERAP1	NOC4L	RPLP1	NOP58	RPS23	DARS	TRIM33	RBBP6	HAUS8	POLR1C	RPS27A	NUS1	EEF1E1
SETDB1	DDX49	ANAPC10	TRNT1	GAPDH	EIF1AD	RRP36	EXOSC5	RFT1	MIS12	LENG8	SNAPC5	ATR	PNO1	WDR18	RAD17	ENO1	GPKOW	ZNF296
MORC3	TSR2	RARS	USP5	POLR3H	EIF2B2	TSR1	NELFCD	ESF1	RPS19BP1	PSME2	DDX27	DHX35	CDC23	DYNLRB1	YARS	NMT1	TUBA1B	KRTAP4-2
MPHOSPH8	FARSB	DKC1	CCDC84	PKM	RPS19	SRP14	ZNF513	WDR43	DDX47	PELP1	RAE1	TEX10	RPL4	GRAMD4	RGS19	DBR1	RAB8B	POLR1B
ATP6V0C	EEF2	ARMC7	NOP16	EIF5	RPL12	CINP	WDR12	RIOK1	KRT80	TWISTNB	DSN1	RPL7	TAF1	EIF2B1	RRP1	RPL24	MRM1	MRTO4
ATP6V1D	DCTN2	GUK1	EEF1A1	CDK9	SBNO1	TUBB	XRN2	GTF2H3	NOP10	RPS21	TPI1	RPS9	MAK16	EIF2B5	COX6B2	BNIP1	AP2S1	RCC1
ATP6V1C1	UTP23	POLR3C	ATRIP	GPN3	DCTN5	DDX10	FCF1	PAK1IP1	NAA25	CEBPZ	GEMIN5	FAU	BAP1	SNRNP25	DHPS	BRAT1	C3orf17	PDCD2
ATP6V1A	IARS	DDX46	EIF6	PPA1	EXOSC7	POLR3B	GGPS1	UXT	PRSS8	RBMX2	EXOSC8	KRI1	YRDC	BRIX1	DDX52	RBM28	ILF3	
ATP6AP2	AATF	GTF2H2	WDR25	GTF2B	TARS	ZDHHC15	C1orf109	EIF2B3	TBP	MESP2	PTPRA	RPE	NELFB	ERI2	SEMA6B	KCTD13	HYI	
ATP2A2	ARL2	NPLOC4	EIF2B4	EIF5A	WDR36	ZNF259	EIF3J	KCMF1	DNTTIP2	RPL35A	CENPN	POP7	WDR5	TRMT112	KPNA6	KANSL2	MAPK8IP3	

d

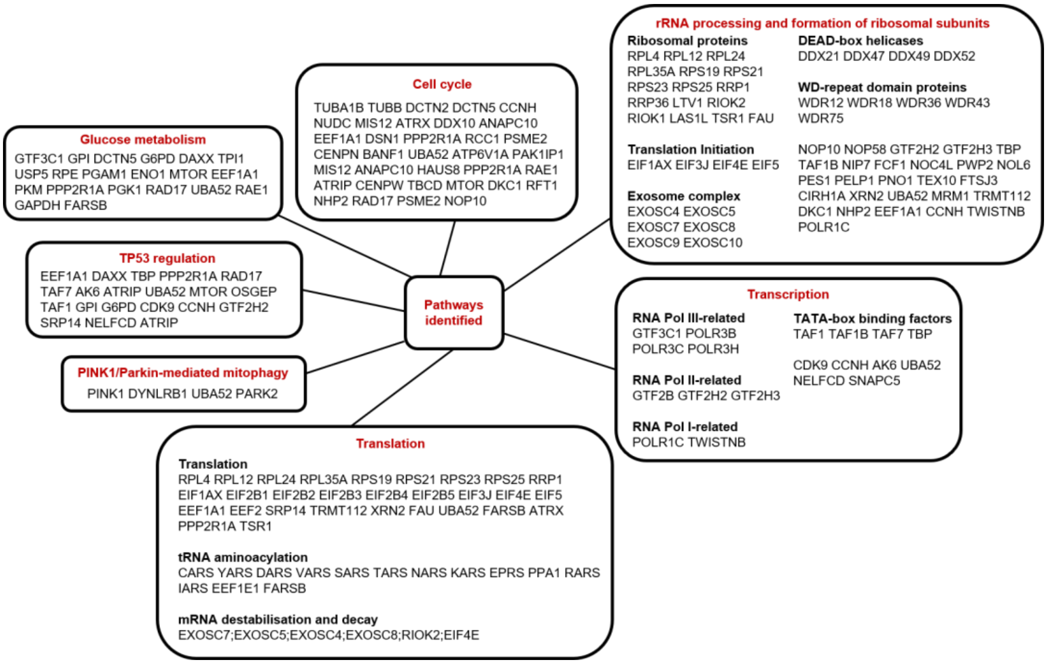

e

Dataset	p-value	sgRNA count	Fold change
#2	2.75E-07	7	8.0095
#4	0.0015577	6	6.3368

Supplementary Figure S2

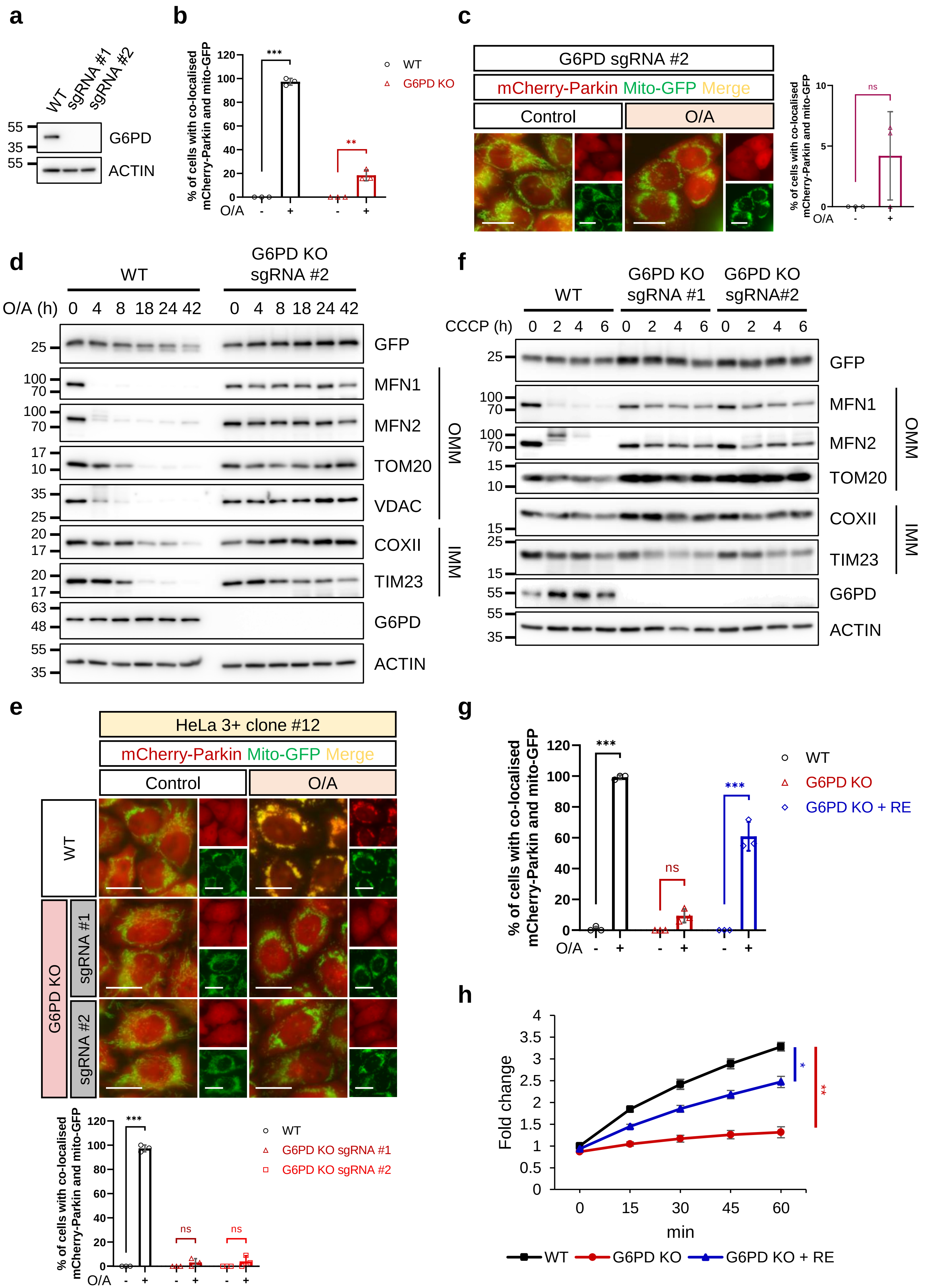

Supplementary Figure S3

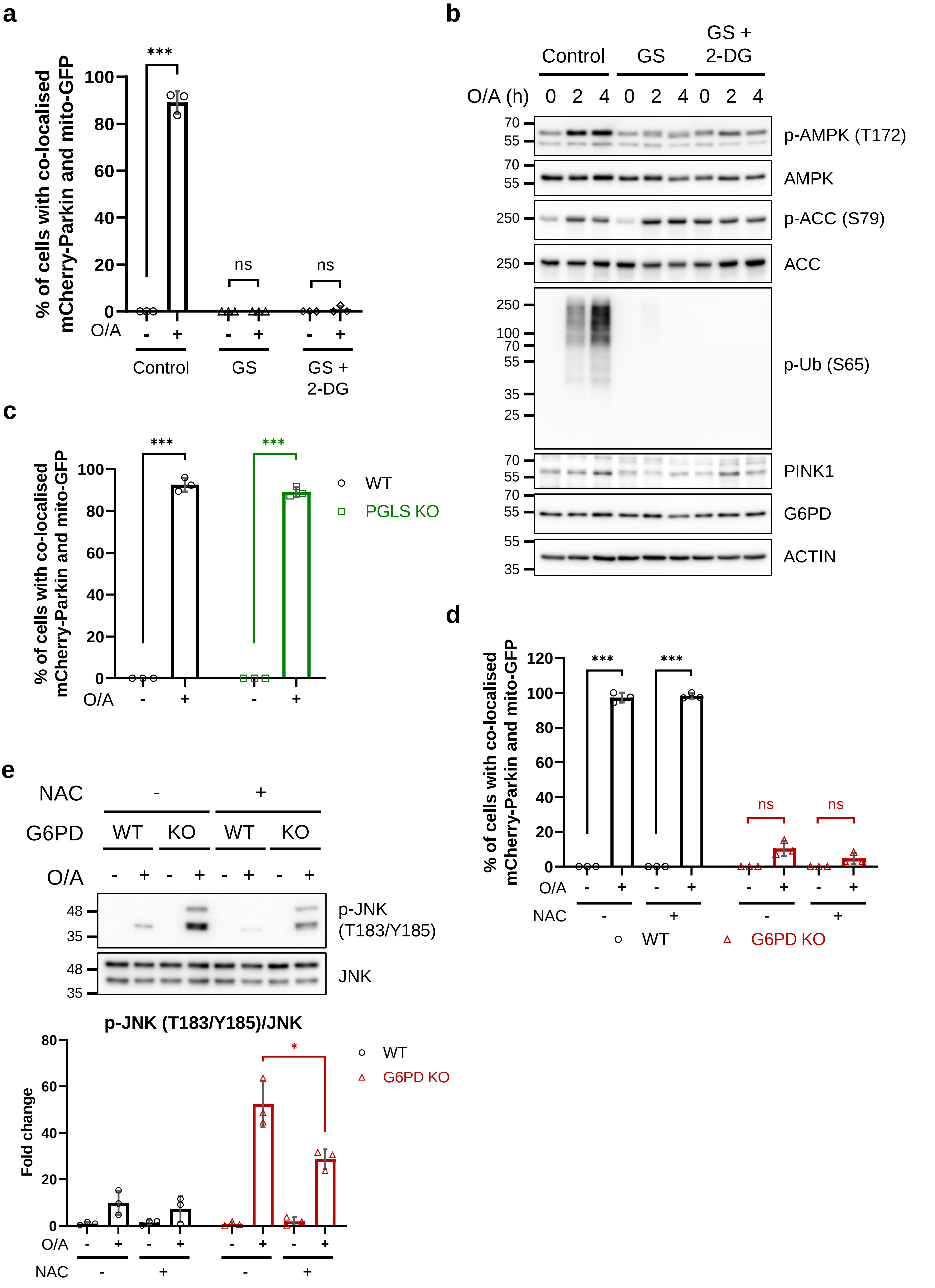

Supplementary Figure S4

a

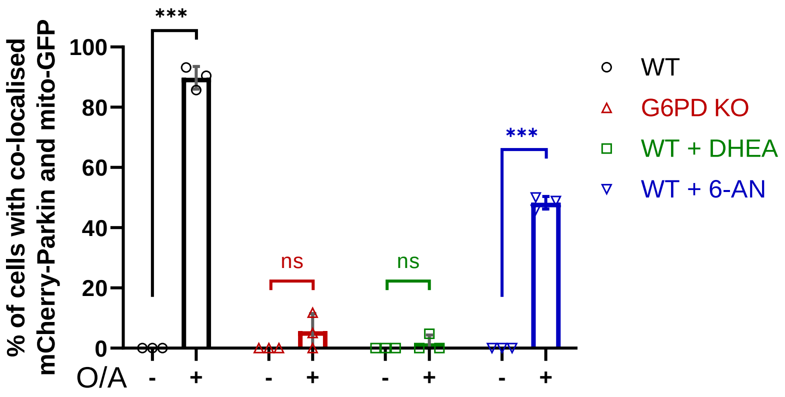

b

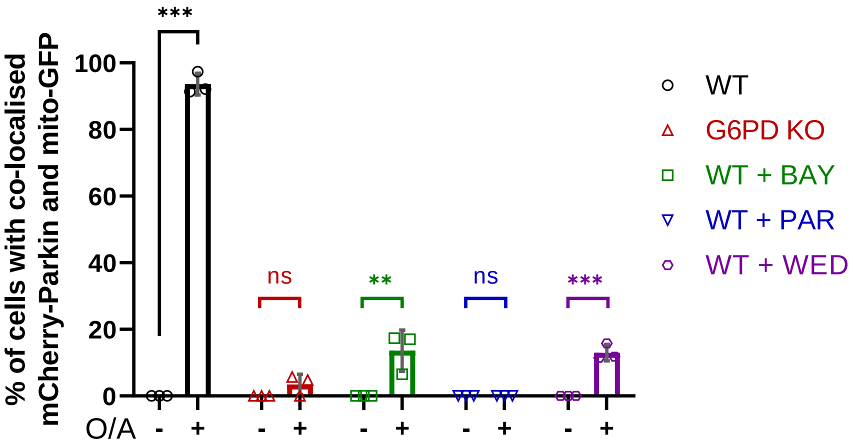

c

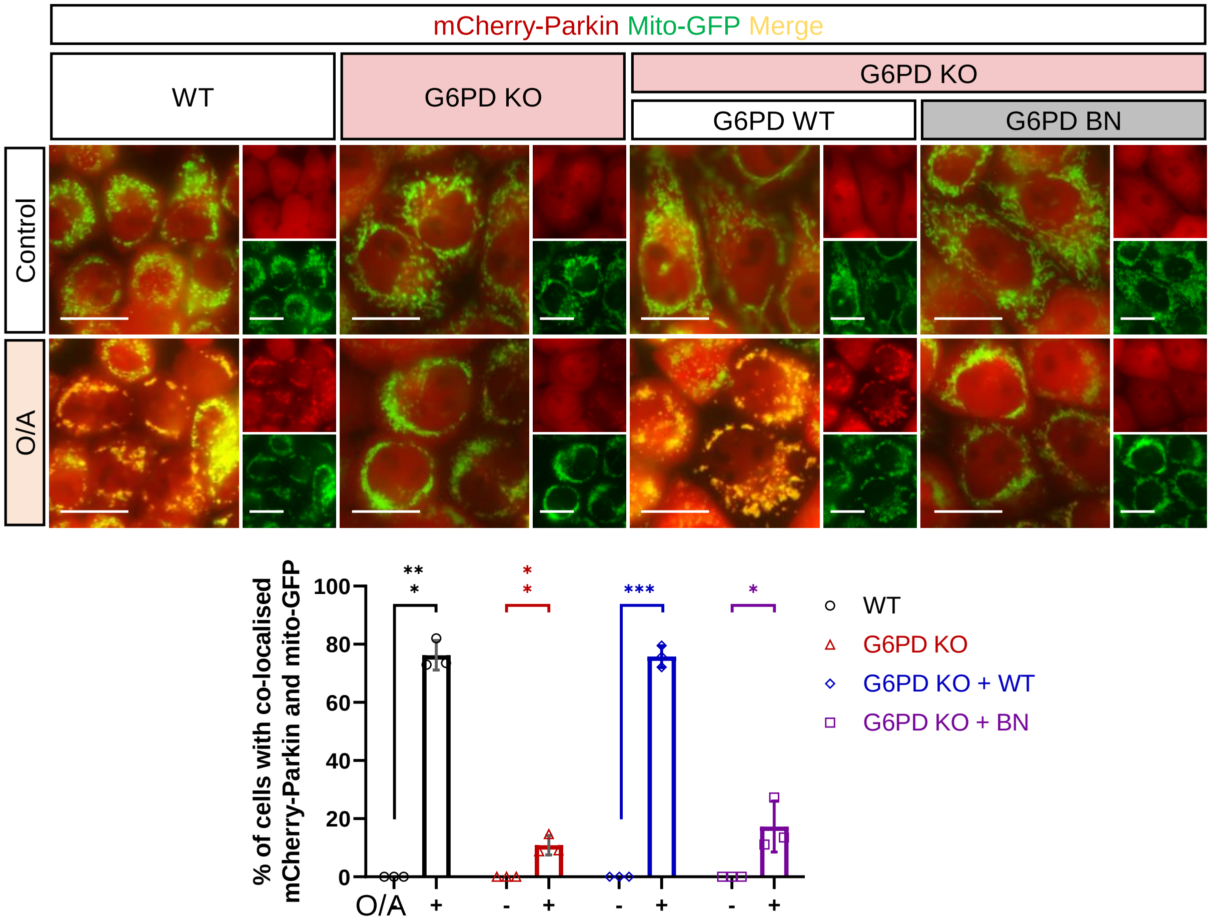

Supplementary Figure S5

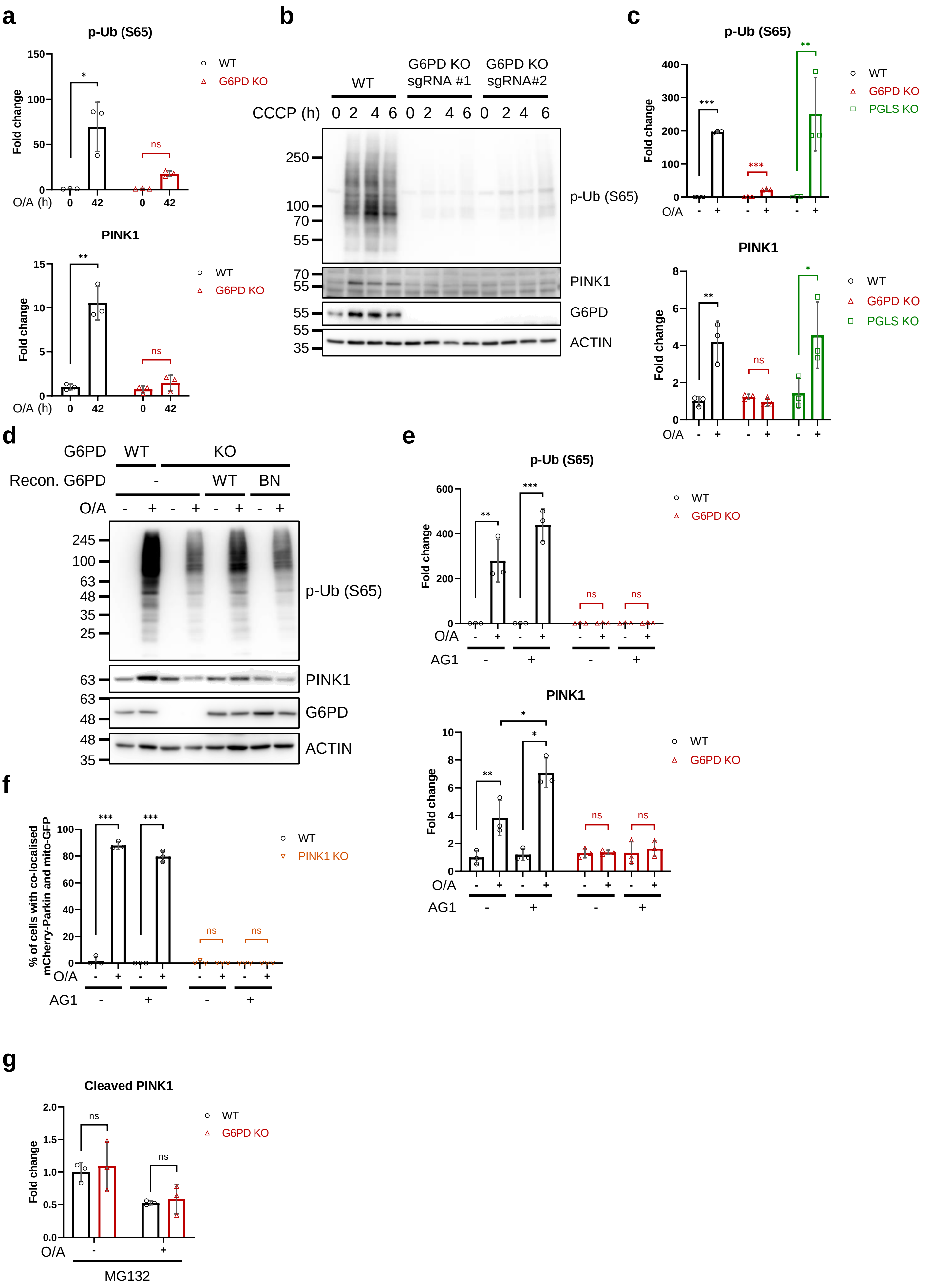

Supplementary Figure S6

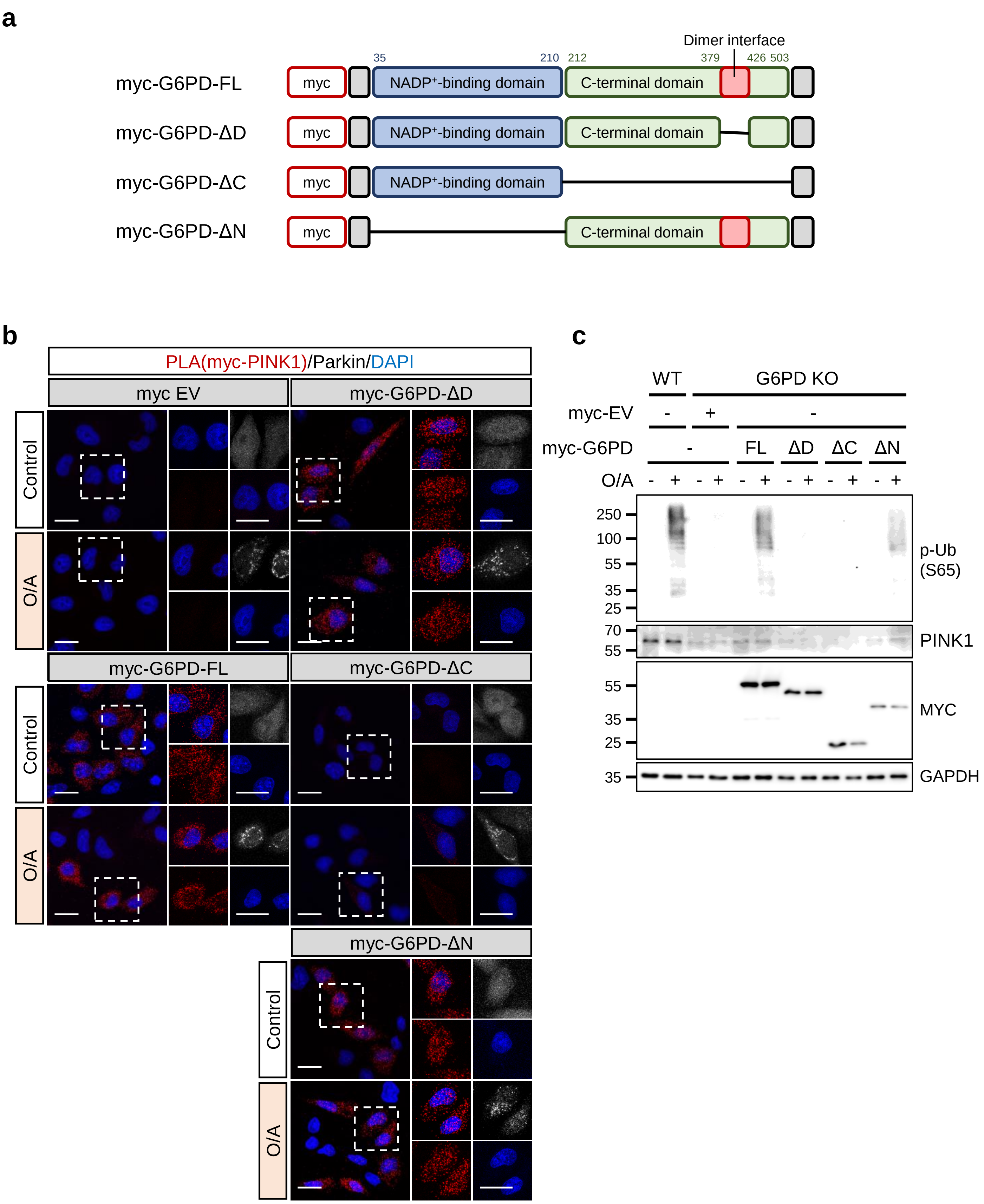

Supplementary Figure S7

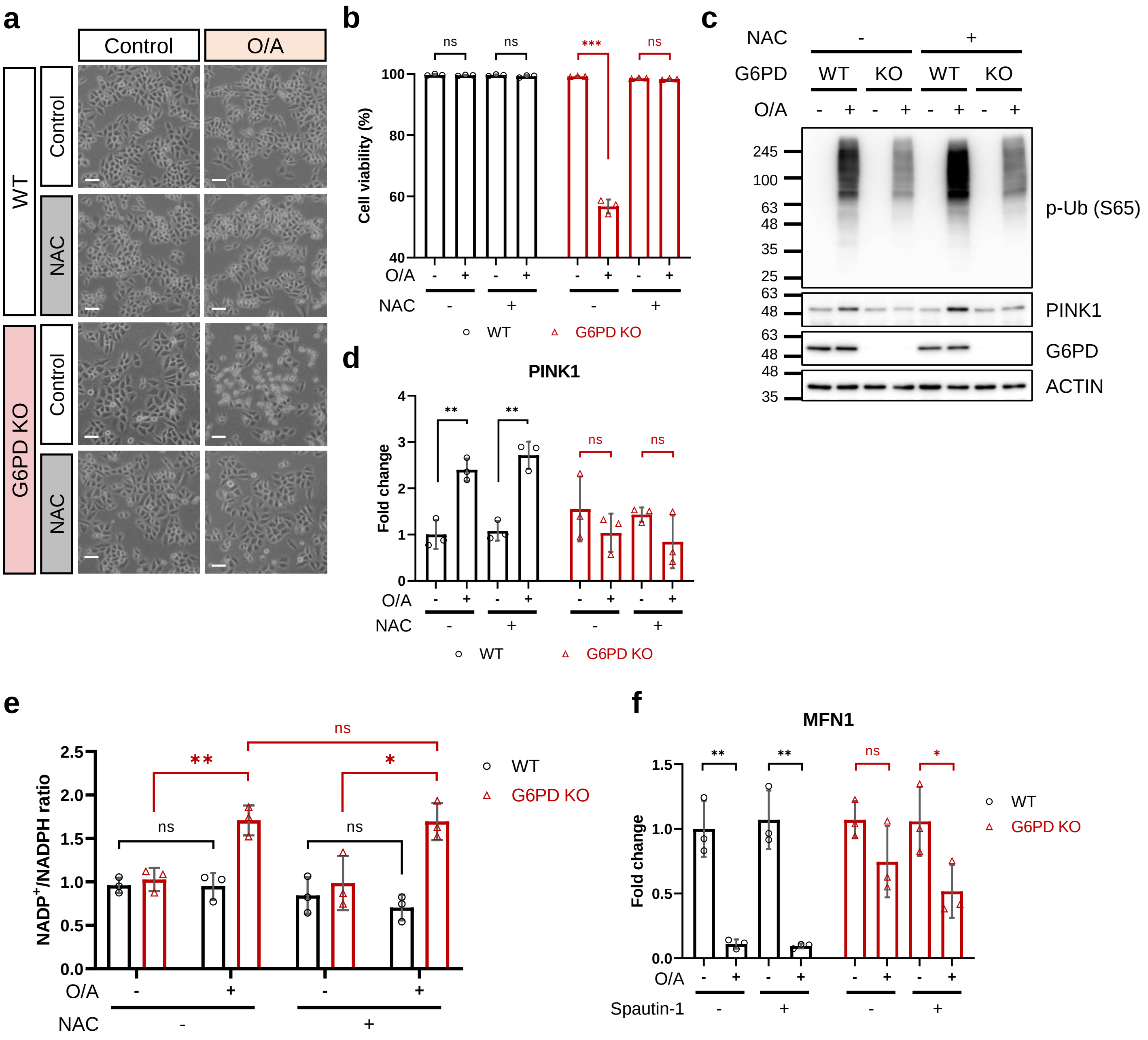

Supplementary Table S1. Primer sequences for creation of G6PD mutants.

**Mutant**	**Primer sequence**
FLAG-G6PD	Forward:<br>5’- AGGATGACGACGATAAGGCAGAGCAGGT GGC-3’<br>Reverse:<br>5’-CGTCGTCATCCTTGTAATCCATGGGAATTC AATCGATAGAACCGA-3’
myc-G6PD	Forward:<br>5’-GAAACTCATCTCTGAAGAGGATCTGGCAGA GCAGGTGGCC-3’<br>Reverse:<br>5’-CAGAGATGAGTTTCTGCTCCATGGGAATTC AATCGATAGAACCGAG-3’
G6PD Bangkok noi (BN)	Forward:<br>5’-AGTGGGTTGCCAGTATGAGGGCAC-3’<br>Reverse:<br>5’-GTGCCCTCATACTGGCAACCCACT-3’
myc-G6PD-ΔD	Forward:<br>5’-CCGGCGACAACAGATACAAGAACGTGAA GCTCC-3’<br>Reverse:<br>5’-ATCTGTTGTCGCCGGCCACATCATG-3’
myc-G6PD-ΔC	Forward:<br>5’-AGAACCTCGAGGGCACCTACAAGTGGGTG-3’<br>Reverse:<br>5’-TGCCCTCGAGGTTCTGCACCATCTCCTTG-3’
myc-G6PD-ΔN	Forward:<br>5’-ACATATTCCTCATGGTGCTGAGATTTGCC-3’<br>Reverse:<br>5’-CCATGAGGAATATGTGTGTATCCGACTGAT GG-3’

Supplementary Table S2. sgRNA sequences for creation of knockout cell lines.

**Gene**	**sgRNA sequence**
PINK1	5’-CACATCAGGGTAGTCGACCA-3’
G6PD (sgRNA #1)	5’-TACCGCATCGACCACTACCT-3’
G6PD (sgRNA #2)	5’-ACGGGCATAGCCCACGATGA-3’
PGLS	5’-AGAGCACGTACGGCCTCTAC-3’

Supplementary Table S3. Barcode primers used to prepare NGS libraries.

**Primer**	**Sequence**
NGS library sgRNA barcode PCR primer forward	5’-CAAGCAGAAGACGGCATACGAGATCnnnnnnTTTCTTGGGTAGTTTGCAGTTTT-3’ (“n” denotes the sample-specified barcode sequence)
NGS library sgRNA barcode PCR primer reverse	5’-AATGATACGGCGACCACCGAGATCTACACnnnnnnnnCACCGACTCGGTGCCACTTTT-3’ (“n” denotes the sample-specified barcode sequence)
