# Supplementary material for: Glucose-6-phosphate dehydrogenase regulates mitophagy by maintaining PINK1 stability
Source: Life Metab. 2024 Dec 13;4(1):loae040. doi: 10.1093/lifemeta/loae040 (PMC11749863; doi:10.1093/lifemeta/loae040)
Supplement: loae040_suppl_Supplementary_Tables_S1-S3 [file loae040_suppl_supplementary_tables_s1-s3.pdf]

Supplementary Table S1. Primer sequences for creation of G6PD mutants.

**Mutant**	**Primer sequence**
FLAG-G6PD	Forward:<br>5’- AGGATGACGACGATAAGGCAGAGCAGGTGGC-3’<br>Reverse:<br>5’-CGTCGTCATCCTTGTAATCCATGGGAATTCAATCGATAGAACCGA-3’
myc-G6PD	Forward:<br>5’-GAAACTCATCTCTGAAGAGGATCTGGCAGAGCAGGTGGCC-3’<br>Reverse:<br>5’-CAGAGATGAGTTTCTGCTCCATGGGAATTCAATCGATAGAACCGAG-3’
G6PD Bangkok noi (BN)	Forward:<br>5’-AGTGGGTTGCCAGTATGAGGGCAC-3’<br>Reverse:<br>5’-GTGCCCTCATACTGGCAACCCACT-3’
myc-G6PD-ΔD	Forward:<br>5’-CCGGCGACAACAGATACAAGAACGTGAAGCTCC-3’<br>Reverse:<br>5’-ATCTGTTGTCGCCGGCCACATCATG-3’
myc-G6PD-ΔC	Forward:<br>5’-AGAACCTCGAGGGCACCTACAAGTGGGTG-3’<br>Reverse:<br>5’-TGCCCTCGAGGTTCTGCACCATCTCCTTG-3’
myc-G6PD-ΔN	Forward:<br>5’-ACATATTCCTCATGGTGCTGAGATTTGCC-3’<br>Reverse:<br>5’-CCATGAGGAATATGTGTGTATCCGACTGATGG-3’

Supplementary Table S2. sgRNA sequences for creation of knockout cell lines.

**Gene**	**sgRNA sequence**
PINK1	5’-CACATCAGGGTAGTCGACCA-3’
G6PD (sgRNA #1)	5’-TACCGCATCGACCACTACCT-3’
G6PD (sgRNA #2)	5’-ACGGGCATAGCCCACGATGA-3’
PGLS	5’-AGAGCACGTACGGCCTCTAC-3’

Supplementary Table S3. Barcode primers used to prepare NGS libraries.

**Primer**	**Sequence**
NGS library sgRNA barcode PCR primer forward	5’-CAAGCAGAAGACGGCATACGAGATCnnnnnnTTTCTTGGGTAGTTTGCAGTTTT-3’ (“n” denotes the sample-specified barcode sequence)
NGS library sgRNA barcode PCR primer reverse	5’-AATGATACGGCGACCACCGAGATCTACACnnnnnnnnCACCGACTCGGTGCCACTTTT-3’ (“n” denotes the sample-specified barcode sequence)
